# Global burden of liver cirrhosis and other chronic liver diseases caused by specific etiologies from 1990 to 2019

**DOI:** 10.1186/s12889-024-17948-6

**Published:** 2024-02-03

**Authors:** Xiao-Ning Wu, Feng Xue, Nan Zhang, Wei Zhang, Jing-Jing Hou, Yi Lv, Jun-Xi Xiang, Xu-Feng Zhang

**Affiliations:** 1 Department of Hepatobiliary Surgery, Institute of Advanced Surgical Technology and Engineering, The First Affiliated Hospital of Xi’an Jiaotong University, 710061 Xi’an, Shaanxi Province China; 2 National-Local Joint Engineering Research Center for Precision Surgery & Regenerative Medicine, Xi’an Jiaotong University, Xi’an, Shaanxi Province China

**Keywords:** Liver cirrhosis, Chronic liver disease, Global burden of disease 2019, Mortality, Incidence

## Abstract

**Background:**

This study aimed to assess the global, regional, and national burden of liver cirrhosis and other chronic liver diseases between 1990 and 2019, considering five etiologies (hepatitis B, hepatitis C, alcohol use, NAFLD and other causes), age, gender, and sociodemographic index (SDI).

**Methods:**

Data on liver cirrhosis and other chronic liver diseases mortality, incidence, and disability-adjusted life years (DALYs) were collected from the Global Burden of Diseases, Injuries, and Risk Factors (GBD) Study 2019.

**Results:**

In 2019, liver cirrhosis and other chronic liver diseases accounted for 1,472,011 (95% UI 1,374,608-1,578,731) deaths worldwide, compared to 1,012,975 (948,941-1,073,877) deaths in 1990. Despite an increase in absolute deaths, the age-standardized death rate declined from 24.43 (22.93–25.73) per 100,000 population in 1990 to 18.00 (19.31–16.80) per 100,000 population in 2019. Eastern sub-Saharan Africa exhibited the highest age-standardized death rate (44.15 [38.47–51.91] per 100,000 population), while Australasia had the lowest rate (5.48 [5.05–5.93] deaths per 100,000 population in 2019). The age-standardized incidence rate of liver cirrhosis and other chronic liver diseases attributed to hepatitis B virus has declined since 1990, but incidence rates for other etiologies have increased. Age-standardized death and DALYs rates progressively decreased with higher SDI across different GBD regions and countries. Mortality due to liver cirrhosis and other chronic liver diseases increased with age in 2019, and the death rate among males was estimated 1.51 times higher than that among females globally.

**Conclusion:**

Liver cirrhosis and other chronic liver diseases continues to pose a significant global public health challenge. Effective disease control, prevention, and treatment strategies should account for variations in risk factors, age, gender, and regional disparities.

**Supplementary Information:**

The online version contains supplementary material available at 10.1186/s12889-024-17948-6.

## Introduction

Liver cirrhosis and other chronic liver diseases ranks as the 14th most common cause of death globally, contributing significantly to mortalities and disability-adjusted life years (DALYs) [1, 2]. It represents an advanced stage of various liver diseases such as hepatitis B and C infections, non-alcoholic fatty liver disease, alcohol consumption, autoimmune disorders, and so on [2, 3]. Typically, liver cirrhosis and other chronic liver diseases develops following prolonged inflammation. The mortality and morbidity associated with liver cirrhosis and other chronic liver diseases increase sharply, with a 1-year case-fatality rate reaching up to 80%. Consequently, liver cirrhosis and other chronic liver diseases poses a substantial burden on patients, healthcare systems, and governments worldwide at both global and regional levels [2, 4].

In recent years, there has been extensive research analyzing the incidence and mortality trends of liver cancer, highlighting its significant disease burden [5–8]. However, compared to liver cancer, reports on the trends of liver cirrhosis and other chronic liver diseases in recent years have been relatively less detailed. The majority of studies on liver cirrhosis and other chronic liver diseases aim to investigate its epidemiology, pathological mechanism and severity based on specific causes [9–11]. The impact of liver cirrhosis and other chronic liver diseases varies significantly across geographical locations, genders, races, ethnicities, and socioeconomic classes, and this impact has also undergone substantial changes over time. Although there have been some reports on the incidence and mortality rates of liver cirrhosis and other chronic liver diseases, they either lack the most recent data or do not comprehensively analyze the disease burden of liver cirrhosis and other chronic liver diseases by integrating incidence rates, mortality rates, DALYs rates, along with various etiological factors, geographical regions, gender, age, and SDI [4, 9–13].

To address this gap, we conducted a systematic analysis using the latest available data from the Global Burden of Disease Study (GBD) 2019. This analysis involved calculating the mortalities, incidence cases, and DALYs for liver cirrhosis and other chronic liver diseases across different GBD regions and countries in 2019. Additionally, ASRs and percentage change in ASRs (APC) from 1990 to 2019 were also calculated to facilitate comparisons. Further analysis for liver cirrhosis and other chronic liver diseases was performed by classifying the data according to five specific etiologies (hepatitis B, hepatitis C, alcohol use, NAFLD and other causes), age groups, gender, and sociodemographic index (SDI). This endeavor aimed to provide the most up-to-date and comprehensive insights into the complete details of liver cirrhosis and other chronic liver diseases.

## Methods

The data used in this study were obtained from the Global Health Data Exchange (GHDx) query tool (https://ghdx.healthdata.org), specifically from the Global Burden of Disease Study (GBD) 2019 [14]. The GBD 2019 was conducted under the coordination of the Institute for Health Metrics and Evaluation (IHME) [10]. Detailed information on liver cirrhosis and other chronic liver diseases, including mortality, incidence, and DALYs, stratified by region, country, sex, age, and etiology, was extracted from the GBD 2019 database.

The dataset encompassed 204 countries and territories, which were categorized into 21 regions based on the classification used in the GBD study. These regions include Andean Latin America, Australasia, Caribbean, Central Asia, Central Europe, Central Latin America, Central sub-Saharan Africa, East Asia, Eastern Europe, Eastern sub-Saharan Africa, High-income Asia Pacific, High-income North America, North Africa and Middle East, Oceania, South Asia, Southeast Asia, Southern Latin America, Southern sub-Saharan Africa, Tropical Latin America, Western Europe and Western sub-Saharan Africa. The etiologies of liver cirrhosis and other chronic liver diseases were divided into HBV, HCV, alcohol use, NAFLD, and other causes [14, 15].

In this study, the trends in global liver cirrhosis and other chronic liver diseases were evaluated using age-standardized incidence rate (ASIR), age-standardized mortality rate (ASMR), and age-standardized DALYs rate (ASDR) stratified by five different etiologies. The annual percentage change (APC) was calculated to measure the change in each trend over time. A positive APC value indicated an increasing burden, while a negative value indicated a declining burden. The burden of liver cirrhosis and other chronic liver diseases in 21 GBD regions and 204 countries and territories were described statistically based on specific values categorized by different groups of sex and age. The Socio-demographic Index (SDI) is a composite indicator that reflects the development status of a country or region. It is calculated based on the rankings of per capita income, average educational attainment, and fertility rates in the GBD study. SDI ranges from 0 (worst) to 1 (best) [14, 16]. The 2.5th and 97.5th centiles were used to determine the 95% uncertainty intervals (UIs). To explore the relationship between the burden of liver cirrhosis and other chronic liver diseases (mortality, incidence, DALYs) and SDIs across the 21 regions and 204 countries and territories, smoothing spline models were employed to examine the shape of the association.

The general linear models are commonly used in statistical analysis, requiring the dependent variables to be normally distributed and independent of each other. These models assume homogeneity of variance and are often utilized for comparing multiple repeated measurements of the same continuous variable. On the other hand, the dependent variable in generalized linear models (GLMs) is not limited to a normal distribution and does not place as much emphasis on homogeneity of variance. A key advantage of GLMs is that the independent variable can be either discrete or continuous, allowing for a broader scope of applications. In this article, GLMs were employed to estimate the mixed effects of gender, SDI, and alcohol use on the age-standardized death rate of liver cirrhosis and other chronic liver diseases [17, 18]. The results are presented through a predictive formula derived from this model:

Y = α0 + α1×Gender + α2×SDI + α3×AlcoholUse + α4×SDI&AlcoholUse + α5×Gender&SDI.

In the given model, α0 represents the overall intercept of the predictor variable. α1, α2, α3, α4, α5, and α6 indicate the slopes of the response variable, reflecting the fixed effects. SDI&AlcoholUse denotes the interaction between the two predictor variables, while Gender&SDI represents the same concept. The relative risk (RR) of mortality for males and females can be assessed by calculating with the aforementioned equation. All statistical analyses were conducted using SPSS 26.0 software (IBM SPSS, Chicago, IL, USA). A p-value less than 0.05 was considered statistically significant. Tables and figures were generated using GraphPad 8.0 or Python.

## Results

### Global burden of liver cirrhosis and other chronic liver diseases

**Fig. 1:**
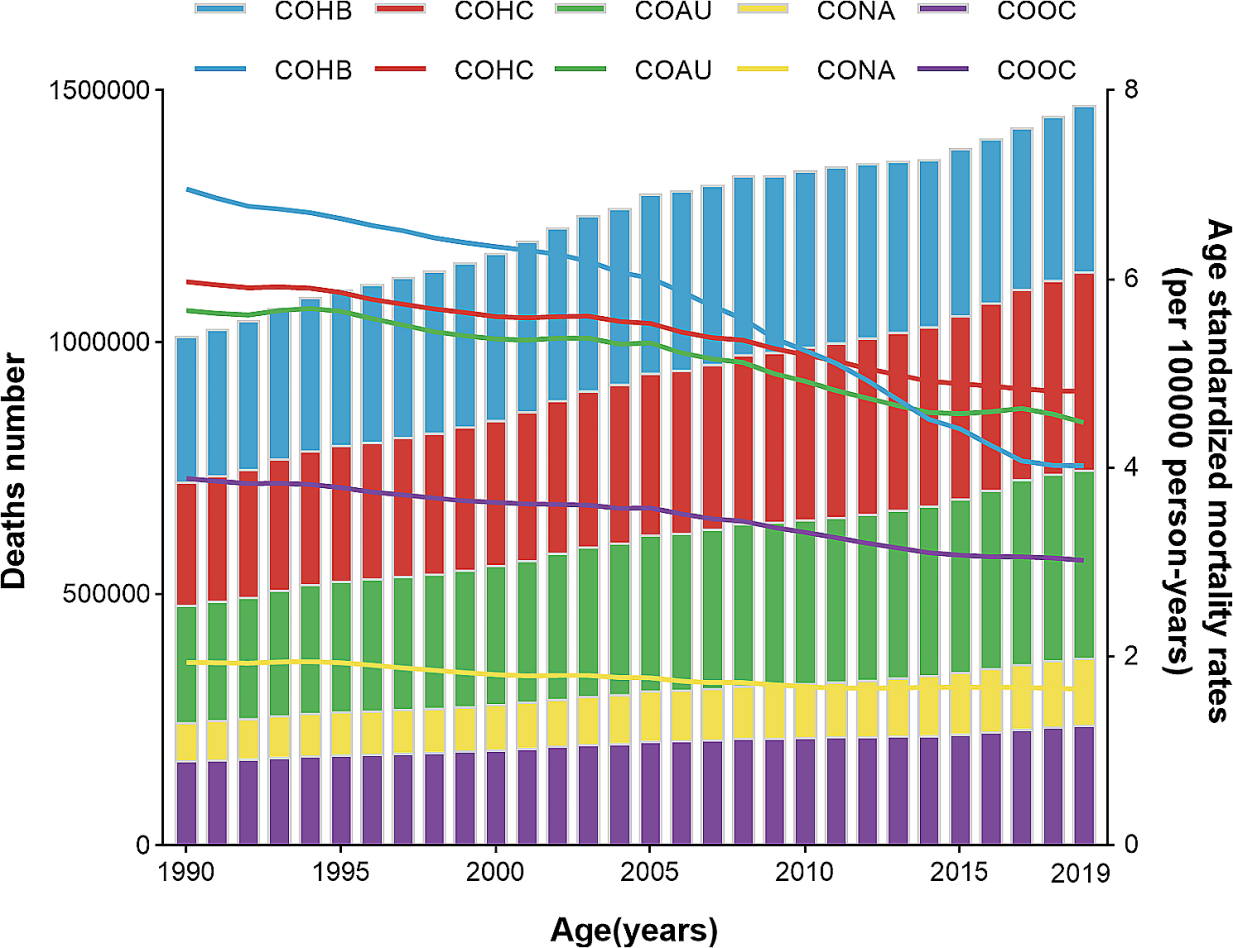
Number of mortality and ASMR at the global level by etiology of liver cirrhosis and other chronic liver diseases, 1990–2019. COHB, liver cirrhosis and other chronic liver diseases due to hepatitis B. COHC, liver cirrhosis and other chronic liver diseases due to hepatitis C. COAU, liver cirrhosis and other chronic liver diseases due to alcohol use. CONA, liver cirrhosis and other chronic liver diseases due to NAFLD. COOC, liver cirrhosis and other chronic liver diseases due to other cause. ASMR, age-standardized mortality rate

**Fig. 2:**
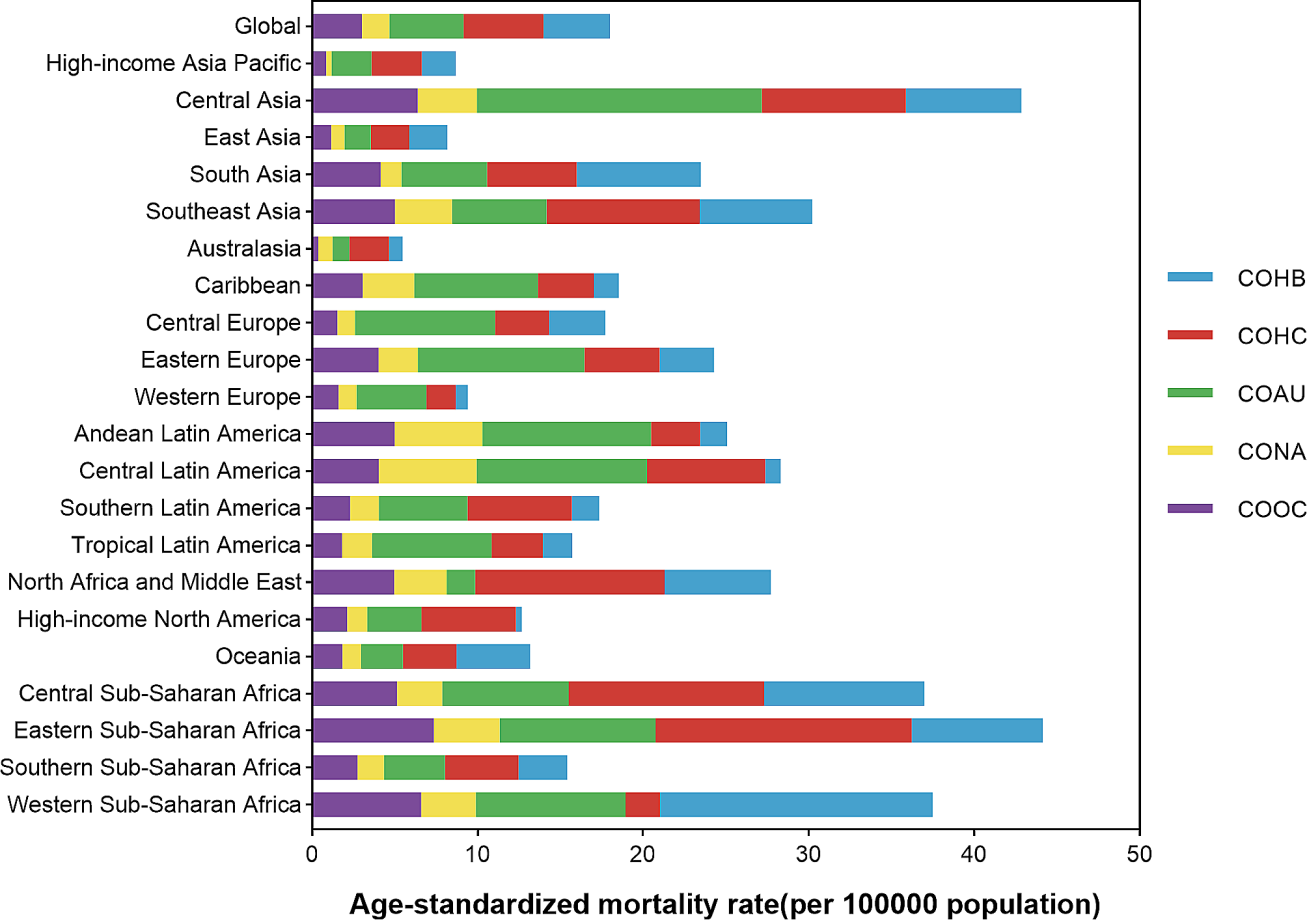
Age-standardized mortality rate for liver cirrhosis and other chronic liver diseases, by region and etiology, 2019. COHB, liver cirrhosis and other chronic liver diseases due to hepatitis B. COHC, liver cirrhosis and other chronic liver diseases due to hepatitis C. COAU, liver cirrhosis and other chronic liver diseases due to alcohol use. CONA, liver cirrhosis and other chronic liver diseases due to NAFLD. COOC, liver cirrhosis and other chronic liver diseases due to other cause

In 2019, there were a total of 1,472,011 deaths (95% UI 1,374,608-1,578,731) caused by liver cirrhosis and other chronic liver diseases worldwide. This marked a significant increase of 45.32% compared to the 1,012,975 deaths (95% UI 948,941-1,073,877) reported in 1990. The ASMR for liver cirrhosis and other chronic liver diseases globally exhibited a notable decrease of 26.32%, declining from 24.43 (22.93–25.73) per 100,000 population in 1990 to 18.00 (16.80-19.31) per 100,000 population in 2019 (Table 1; Figs. 1 and 2). The incidence of liver cirrhosis and other chronic liver diseases in individuals of all ages witnessed a 62.03% increase, rising from 1,274,022 (1,027,186-1,548,534) cases in 1990 to 2,051,553 (1,661,430-2,478,127) cases in 2019. The global ASIR was 25.66 (20.25–31.63) per 100,000 population in 1990, showing a slight decrease to 25.35 (20.78–30.44) per 100,000 population in 2019 (Table 1; Figure S1 and S2). In 2019, liver cirrhosis and other chronic liver diseases resulted in a total of 46,189,415 (43,027,109 − 49,551,291) DALYs, representing a 33.00% increase compared to the 34,727,732 (32,382,957 − 37,132,796) DALYs in 1990. Conversely, the global ASDR exhibited a decreasing trend from 766.07 (718.30-813.11) per 100,000 population in 1990 to 560.43 (521.86-602.02) per 100,000 population in 2019 (Table 1; Figure S3 and S4), indicating a decline of 26.84%.

**Table 1:** Death, incident cases, and disability-adjusted life years (DALYs) for cirrhosis and other chronic liver diseases in 2019 and percentage change in age-standardized rates (ASRs) per 100,000 population from 1990 to 2019 by Global Burden of Disease regions

Characteristics	Death (95% uncertainty interval)			Incidence (95% uncertainty interval)			DALYs (95% uncertainty interval)		
	Counts	ASR per 100,000 population (95% UI)	Percentage change in ASRs per 100,000 population (95% UI)	Counts	ASR per 100,000 population (95% UI)	Percentage change in ASRs per 100,000 population (95% UI)	Counts	ASR per 100,000 population (95% UI)	Percentage change in ASRs per 100,000 population (95% UI)
Global	1,472,011 (1,374,608 to 1,578,731)	18.00 (16.80 to 19.31)	-0.26 (-0.32 to -0.20)	2,051,553 (1,661,430 to 2,478,127)	25.35 (20.78 to 30.44)	-0.01 (-0.04 to 0.03)	46,189,415 (43,027,109 to 49,551,291)	560.43 (521.86 to 602.02)	-0.27 (-0.32 to -0.19)
Sex									
Male	969,067 (899,212 to 1,045,344)	24.81 (23.07 to 26.75)	-0.26 (-0.32 to -0.18)	1,206,124 (964,222 to 1,464,589)	29.67 (23.86 to 35.98)	-0.01 (-0.05 to 0.03)	31,781,079 (29,366,251 to 34,438,138)	783.31 (723.85 to 849.07)	-0.26 (-0.32 to -0.18)
Female	502,944 (459,201 to 550,914)	11.70 (10.68 to 12.81)	-0.28 (-0.34 to -0.20)	845,429 (687,513 to 1,016,947)	20.91 (17.22 to 25.15)	-0.01 (-0.04 to 0.04)	14,408,336 (13,159,620 to 15,759,495)	343.96 (313.74 to 376.74)	-0.29 (-0.36 to -0.21)
Regions									
Andean Latin America	14,051 (11,229 to 17,327)	25.08 (20.08 to 30.92)	-0.20 (-0.40 to 0.06)	19,973 (17,614 to 22,665)	32.25 (28.40 to 36.69)	0.39 (0.28 to 0.50)	373,058 (294,137 to 465,085)	639.08 (506.08 to 795.50)	-0.31 (-0.49 to -0.06)
Australasia	2511 (2297 to 2731)	5.48 (5.05 to 5.93)	-0.22 (-0.28 to -0.16)	3314 (2795 to 3818)	10.26 (8.75 to 11.72)	-0.08 (-0.14 to -0.01)	63,252 (58,693 to 67,741)	153.41 (142.81 to 164.14)	-0.25 (-0.30 to -0.19)
Caribbean	9545 (7776 to 11,408)	18.52 (15.04 to 22.19)	-0.20 (-0.33 to -0.05)	10,842 (9201 to 12,553)	21.85 (18.56 to 25.08)	0.08 (0.03 to 0.13)	278,278 (219,587 to 341,080)	545.75 (429.14 to 672.58)	-0.22 (-0.36 to -0.08)
Central Asia	33,909 (30,488 to 37,737)	42.86 (38.53 to 47.51)	0.55 (0.39 to 0.73)	58,160 (51,498 to 64,782)	59.06 (52.30 to 66.01)	0.80 (0.73 to 0.89)	1,173,144 (1,054,257 to 1,309,148)	1318.24 (1187.00 to 1467.06)	0.55 (0.40 to 0.74)
Central Europe	33,596 (29,317 to 37,899)	17.71 (15.42 to 20.04)	-0.21 (-0.31 to -0.11)	37,555 (33,208 to 41,704)	29.11 (25.85 to 32.45)	-0.11 (-0.15 to -0.08)	964,512 (839,764 to 1,091,670)	554.84 (483.06 to 627.72)	-0.22 (-0.31 to -0.12)
Central Latin America	68,062 (58,609 to 78,330)	28.32 (24.45 to 32.59)	-0.22 (-0.32 to -0.10)	106,174 (85,022 to 127,867)	40.76 (32.74 to 48.98)	-0.05 (-0.12 to 0.04)	2,031,944 (1,740,913 to 2,343,602)	816.65 (700.77 to 940.38)	-0.26 (-0.37 to -0.15)
Central Sub-Saharan Africa	22,757 (17,088 to 29,114)	36.98 (27.96 to 47.37)	-0.29 (-0.47 to -0.08)	30,833 (25,653 to 36,783)	26.97 (21.49 to 33.11)	0.19 (0.11 to 0.30)	843,049 (629,421 to 1,106,949)	1089.72 (821.03 to 1392.66)	-0.28 (-0.47 to -0.04)
East Asia	164,719 (140,148 to 191,748)	8.18 (7.01 to 9.46)	-0.58 (-0.65 to -0.48)	424,355 (321,444 to 529,848)	22.51 (17.71 to 27.56)	-0.18 (-0.24 to -0.08)	4,698,613 (3,982,096 to 5,513,225)	227.88 (193.82 to 266.21)	-0.61 (-0.68 to -0.51)
Eastern Europe	72,717 (64,994 to 81,005)	24.29 (21.67 to 27.04)	1.29 (1.04 to 1.55)	66,873 (47,654 to 90,004)	31.27 (23.38 to41.10)	0.57 (0.36 to 0.80)	2,575,334 (2,298,784 to 2,874,694)	919.51 (820.66 to 1026.76)	1.71 (1.43 to 2.02)
Eastern Sub-Saharan Africa	76,980 (66,309 to 91,419)	44.15 (38.47 to 51.91)	-0.25 (-0.39 to -0.07)	86,022 (67,755 to 105,518)	27.15 (19.80 to 35.41)	0.07 (0.03 to 0.13)	2,608,125 (2,191,259 to 3,151,158)	1178.42 (1015.50 to 1402.45)	-0.28 (-0.44 to -0.06)
High-income Asia Pacific	36,888 (32,213 to 41,082)	8.69 (7.87 to 9.38)	-0.58 (-0.61 to -0.54)	50,826 (41,597 to 60,069)	25.15 (21.29 to 29.18)	-0.36 (-0.41 to -0.29)	776,896 (717,257 to 824,271)	230.17 (216.01 to 242.91)	-0.62 (-0.64 to -0.58)
High-income North America	72,738 (69,312 to 75,464)	12.67 (12.16 to 13.11)	0.04 (0.01 to 0.08)	98,636 (81,095 to 116,829)	25.62 (21.47 to 30.08)	0.09 (-0.01 to 0.23)	1,955,333 (1,892,680 to 2,014,258)	371.36 (360.22 to 381.81)	0.00 (-0.03 to 0.03)
North Africa and Middle East	109,706 (81,390 to 135,204)	27.73 (21.06 to 33.88)	-0.37 (-0.53 to -0.21)	160,139 (133,538 to 190,699)	28.68 (23.58 to 34.88)	0.21 (0.14 to 0.29)	2,878,426 (2,126,424 to 3,565,003)	616.80 (457.71 to 761.65)	-0.37 (-0.54 to -0.20)
Oceania	1152 (905 to 1445)	13.19 (10.56 to 16.35)	-0.20 (-0.38 to -0.01)	1123 (935 to 1323)	8.50 (7.05 to 10.00)	-0.13 (-0.18 to -0.08)	45,977 (35,984 to 57,692)	440.77 (347.93 to 550.45)	-0.21 (-0.39 to 0.00)
South Asia	348,393 (306,924 to 404,845)	23.49 (20.74 to 27.13)	-0.24 (-0.35 to -0.10)	413,984 (299,626 to 539,338)	22.98 (16.68 to 29.92)	0.33 (0.25 to 0.41)	12,436,281 (10,954,316 to 14,414,136)	750.60 (662.20 to 867.89)	-0.24 (-0.35 to -0.10)
Southeast Asia	186,154 (165,361 to 207,697)	30.21 (26.88 to 33.49)	-0.27 (-0.38 to -0.15)	181,538 (142,578 to 219,743)	24.76 (19.50 to 30.00)	-0.06 (-0.13 to 0.04)	5,915,348 (5,207,218 to 6,642,922)	866.96 (765.10 to 970.68)	-0.33 (-0.42 to -0.22)
Southern Latin America	14,153 (13,221 to 15,117)	17.34 (16.22 to 18.49)	-0.23 (-0.28 to -0.18)	22,198 (19,634 to 24,756)	30.50 (27.07 to 34.08)	0.18 (0.10 to 0.26)	372,367 (351,788 to 394,658)	471.63 (446.43 to 499.36)	-0.30 (-0.34 to -0.26)
Southern Sub-Saharan Africa	9230 (8229 to 10,319)	15.43 (13.82 to 17.16)	-0.32 (-0.49 to -0.14)	12,183 (9241 to 15,402)	15.57 (11.70 to 19.87)	-0.19 (-0.23 to -0.14)	310,793 (272,943 to 353,505)	458.73 (405.32 to 516.57)	-0.36 (-0.51 to -0.20)
Tropical Latin America	38,778 (36,545 to 41,257)	15.72 (14.81 to 16.75)	-0.35 (-0.39 to -0.30)	50,186 (36,235 to 65,544)	19.79 (14.41 to 25.57)	-0.24 (-0.30 to -0.16)	1,193,339 (1,134,407 to 1,261,299)	473.87 (449.62 to 501.06)	-0.39 (-0.43 to -0.35)
Western Europe	77,225 (72,044 to 82,464)	9.41 (8.93 to 9.99)	-0.41 (-0.44 to -0.37)	116,117 (102,873 to 129,245)	24.45 (21.91 to 26.93)	-0.19 (-0.22 to -0.16)	1,832,992 (1,752,143 to 1,930,379)	260.88 (250.64 to 273.57)	-0.44 (-0.47 to -0.41)
Western Sub-Saharan Africa	78,736 (61,695 to 99,765)	37.50 (30.28 to 46.47)	-0.29 (-0.45 to -0.11)	100,452 (80,812 to 121,906)	26.22 (19.92 to 33.23)	0.10 (0.04 to 0.17)	2,862,344 (2,196,425 to 3,688,871)	1052.18 (823.19 to 1337.56)	-0.30 (-0.45 to -0.11)

**Fig. 3:**
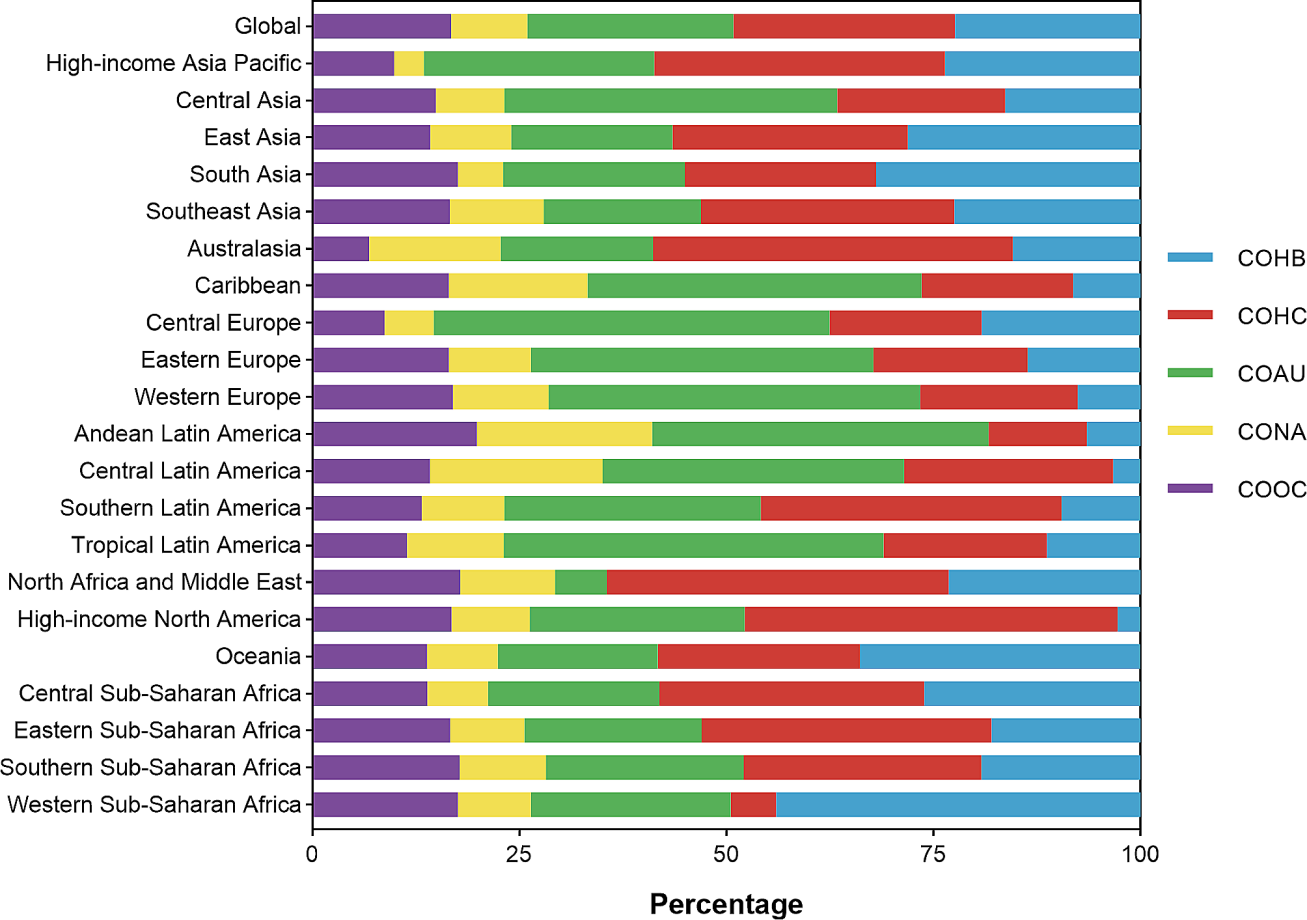
Contribution of COHB, COHC, COAU, CONA, and COOC to liver cirrhosis and other chronic liver diseases mortality, both sexes, globally and by region, 2019. COHB, liver cirrhosis and other chronic liver diseases due to hepatitis B. COHC, liver cirrhosis and other chronic liver diseases due to hepatitis C. COAU, liver cirrhosis and other chronic liver diseases due to alcohol use. CONA, liver cirrhosis and other chronic liver diseases due to NAFLD. COOC, liver cirrhosis and other chronic liver diseases due to other cause

In 2019, hepatitis B accounted for approximately 22.36% of all liver cirrhosis and other chronic liver diseases-related deaths in individuals of all ages. Hepatitis C was responsible for 26.75% of these deaths, while alcohol use contributed to 24.90%. Non-alcoholic steatohepatitis (NASH) caused 9.20% of the mortalities, and other causes accounted for 16.78% (Fig. 3).

Specifically, there were 331,266 (278,542 − 392,126) deaths attributed to liver cirrhosis and other chronic liver diseases caused by hepatitis B (COHB) in 2019, representing a 14.36% increase from 1990 [289,682 (249,026–331,355)]. However, the ASMR for liver cirrhosis and other chronic liver diseases caused by hepatitis B decreased by 42.10% during this period, dropping from 6.96 (6.00-7.95) per 100,000 population in 1990 to 4.03 (3.39–4.76) per 100,000 population in 2019, and HBV infection now ranks as the third leading cause for ASMR of liver cirrhosis and other chronic liver diseases. For liver cirrhosis and other chronic liver diseases caused by hepatitis C (COHC), there were 245,022 (208,652 − 282,509) deaths in 1990 and 395,022 (335,832 − 458,584) deaths in 2019. The ASMR for COHC declined by 19.26% from 5.97 (5.09–6.90) per 100,000 population in 1990 to 4.82 (4.09–5.57) per 100,000 population in 2019. Liver cirrhosis and other chronic liver diseases resulting from alcohol use (COAU) caused 232,949 (197,746 − 273,817) deaths in 1990 and 371,964 (314,703 − 438,425) deaths in 2019. The ASMR for COAU decreased by 20.99% worldwide, going from 5.67 (4.80–6.64) per 100,000 population in 1990 to 4.48 (3.81–5.28) per 100,000 population in 2019. In 1990, there were 75,957 (54,215 − 101,590) deaths attributed to liver cirrhosis and other chronic liver diseases due to non-alcoholic fatty liver disease (CONA), and this number increased to 134,240 (96,483 − 176,920) deaths in 2019. The ASMR for CONA decreased by 14.43% from 1.94 (1.39–2.59) per 100,000 population in 1990 to 1.66 (1.20–2.17) per 100,000 population in 2019. Moreover, liver cirrhosis and other chronic liver diseases due to other causes (COOC) resulted in 169,363 (137,356 − 206,849) deaths in 1990 and 239,517 (187,991 − 302,873) deaths in 2019. The ASMR for COOC decreased by 22.37%, declining from 3.89 (3.12–4.83) per 100,000 population in 1990 to 3.02 (3.38–3.78) per 100,000 population in 2019 (Figs. 1 and 2).

### Geographic burden of liver cirrhosis and other chronic liver diseases

In 2019, the regions with the top two ASMR for liver cirrhosis and other chronic liver diseases were Eastern Sub-Saharan Africa and Central Asia, with rates of 44.15 (38.47–51.91) and 42.86 (38.53–47.51) per 100,000 population respectively. Central Asia experienced a significant burden of liver cirrhosis and other chronic liver diseases, with ranking first in ASIR among all regions (Fig. 2; Figure S2). It also had the highest ASDR for liver cirrhosis and other chronic liver diseases, with a rate of 1318 (1187–1467) per 100,000 population in 2019 (Figure S4). Central Asia and Eastern Europe consistently showed the most significant increases in ASMR, ASIR, and ASDR from 1990 to 2019 compared to other regions, which experienced varying degrees of decline. North Africa and the Middle East had the most dramatic decrease in ASMR over the past 30 years. High-income Asia Pacific had the largest drop in ASIR, while Eastern Sub-Saharan Africa experienced the largest decline in ASDR from 1990 to 2019.

Hepatitis B was the leading cause of liver cirrhosis and other chronic liver diseases in Western Sub-Saharan Africa, Oceania, and South Asia, accounting for 43.96%, 33.85%, and 31.88% of total liver cirrhosis and other chronic liver diseases deaths in 2019 respectively (Figs. 2 and 3). The highest ASMR for COHB for all ages was observed in Western Sub-Saharan Africa (16.69 [12.69–21.35]), while the lowest was found in East Asia (2.30 [1.87–2.78]). Eastern Sub-Saharan Africa had the highest ASMR for COHC in 2019 (15.46 [12.52–19.04]), while Western Europe had the lowest ASMR for COHC (1.79 [1.41–2.25]). More than 40% of liver cirrhosis and other chronic liver diseases-related deaths caused by hepatitis C were found in high-income regions such as North America, Australasia, and North Africa and the Middle East (Figs. 2 and 3). Central Asia had the highest ASMR for COAU for all ages in 2019 (17.22 [13.83–20.69]), while Australasia had the lowest (1.01 [0.72–1.36]). Mortalities attributed to COAU accounted for over 40% in Central Europe, Tropical Latin America, Western Europe, Eastern Europe, Andean Latin America, Caribbean, and Central Asia (Figs. 2 and 3). The proportion of mortalities for liver cirrhosis and other chronic liver diseases due to NAFLD was lower than that due to other causes in most regions in 2019. Andean Latin America consistently had the highest proportions attributable to NAFLD and other causes of liver cirrhosis and other chronic liver diseases (21.17% and 19.88% respectively) in ASMR. The highest ASMR for CONA was in Central Latin America (5.90 [4.32–7.66]), while Eastern Sub-Saharan Africa had the highest ASMR for COOC in 2019 (7.35 [5.23–9.63]) (Figs. 2 and 3).

### National burden of liver cirrhosis and other chronic liver diseases

**Fig. 4:**
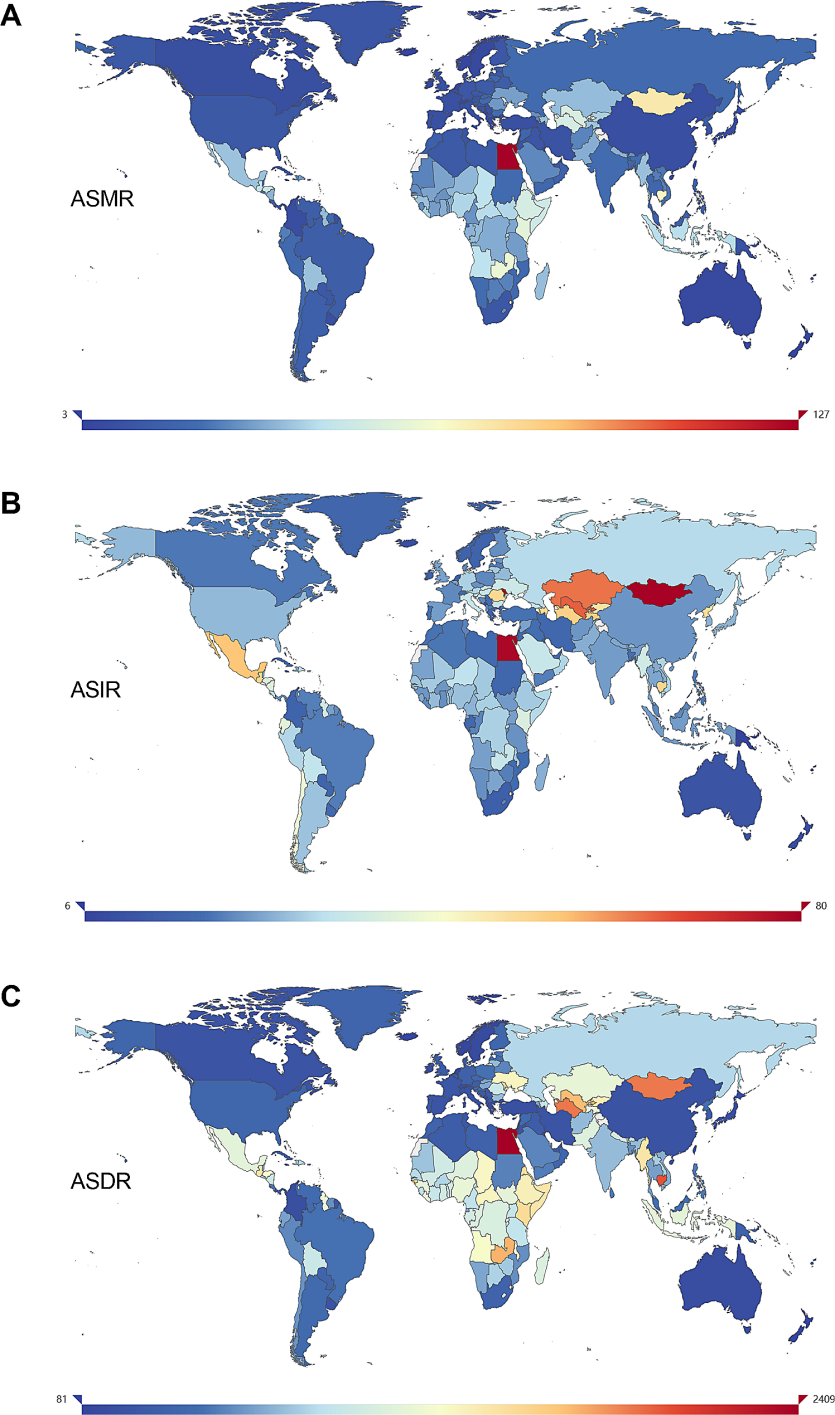
The global age-standardized rate of liver cirrhosis and other chronic liver diseases per 100,000 populations in 2019, by country and territory. (**A**) ASMR in 2019; (**B**) ASIR in 2019; and (**C**) ASDR in 2019. ASMR, age-standardized mortality rate. ASIR, age-standardized incidence rate. ASDR, age-standardized DALYs rate

At the national level, India had the highest number of deaths (270,036 [228,609 − 321,257]) and DALYs (9,635,297 [8,187,694 − 11,300,327]) attributed to liver cirrhosis and other chronic liver diseases. China had the highest number of incident cases (409,693 [308,245–514,354]). In China, the estimated ASMR for liver cirrhosis and other chronic liver diseases ranged from 126.73 to 3.26 per 100,000 population in 2019. In contrast, Singapore had the lowest ASMR and ASDR for liver cirrhosis and other chronic liver diseases, and the lowest ASIR was observed in both Cook Islands and Papua New Guinea (Fig. 4). The largest increases in ASMR and ASDR over the past 30 years were seen in Ukraine, and the biggest increase in ASIR was observed in Kazakhstan. Korea showed the most notable decreases in these rates from 1990 to 2019 (Figure S7). For India, Indonesia, and Pakistan, three heavily populated countries, the annual percentage of change from 1990 to 2019 in the ASMR was (-17% [-32–2%]), (-17% [-32–1%]), and (-16% [-38–21%]), respectively.

In terms of specific etiologies, Egypt had the highest ASMR of COHB in 2019 (32.67 [20.28–47.58]), while Colombia had the lowest (0.22 [0.14–0.34]). Ukraine had the largest increase in ASMR of COHB from 1990 to 2019 (115% [72–166%]), while Korea had the largest decrease (-82% [-85% to -77%]) (Figure S8A and S9A).

Egypt also had the highest ASMR of COHC in 2019 (52.58 [33.36–73.18]), while Iceland had the lowest (0.28 [0.20–0.40]). Ukraine experienced the most significant increase in ASMR of COHC from 1990 to 2019 (231% [168–305%]), while Korea had the largest decrease (-69% [-74% to -61%]) (Figure S10A and S11A).

Mongolia had the highest ASMR of COAU in 2019 (29.25 [21.58–38.89]), while Singapore had the lowest (0.52 [0.36–0.73]). The countries with the greatest increase and decrease in ASMR of COAU were the same as those for COHB and COHC, with percent changes ranging from − 68 to 198% (Figure S12A and S13A).

Regarding CONA and COOC in 2019, Egypt also had the highest ASMR, while Singapore had the lowest ASMR for CONA, and New Zealand had the lowest ASMR for COOC. Ukraine showed the largest increases in ASMR for CONA and COOC, while Korea had the largest decreases (Figure S14A, S15A, S16A, and S17A).

For more detailed information on incident numbers, DALYs, ASIR, ASDR, and annual rates of percent change for different etiologies globally, by region, and by country, please refer to supplementary materials (Figure S7-S17).

### Age and sex on liver cirrhosis and other chronic liver diseases

**Fig. 5:**
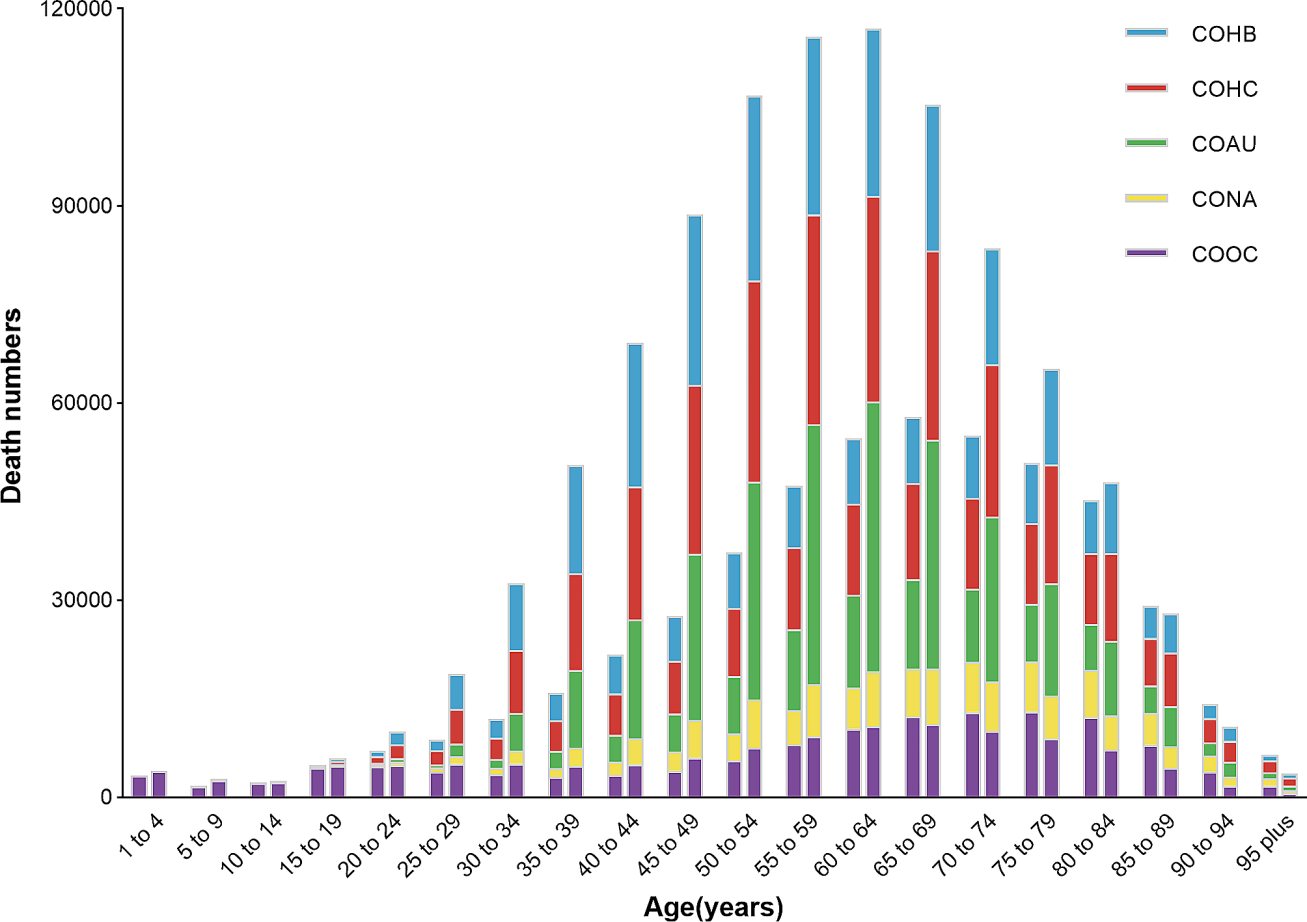
Global liver cirrhosis and other chronic liver diseases mortality by etiology and age for females and males, 2019. For each group, the left column showed case data in female and the right column shows data in male. COHB, liver cirrhosis and other chronic liver diseases due to hepatitis B. COHC, liver cirrhosis and other chronic liver diseases due to hepatitis C. COAU, liver cirrhosis and other chronic liver diseases due to alcohol use. CONA, liver cirrhosis and other chronic liver diseases due to NAFLD. COOC, liver cirrhosis and other chronic liver diseases due to other cause

In 2019, the global number of deaths due to liver cirrhosis and other chronic liver diseases was higher in males than females across all age groups, except for those aged older than 85 years (Fig. 5). This trend was also observed for DALYs, while the incidence of liver cirrhosis and other chronic liver diseases was higher in males only within the age range of 10–49 years (Figure S18 and S19).

The number of mortalities from liver cirrhosis and other chronic liver diseases in 2019 exhibited a sharp increase after the age of 20, reaching its peak at the ages of 60–64 years for males and 65–69 years for females. Subsequently, the number of deaths declined in older age groups. The lowest number of deaths occurred in patients younger than 15 years. The development of liver cirrhosis and other chronic liver diseases caused by different etiologies exhibited age-related and gender-specific patterns (Fig. 5).

### Burden of liver cirrhosis and other chronic liver diseases by sociodemographic index

**Fig. 6:**
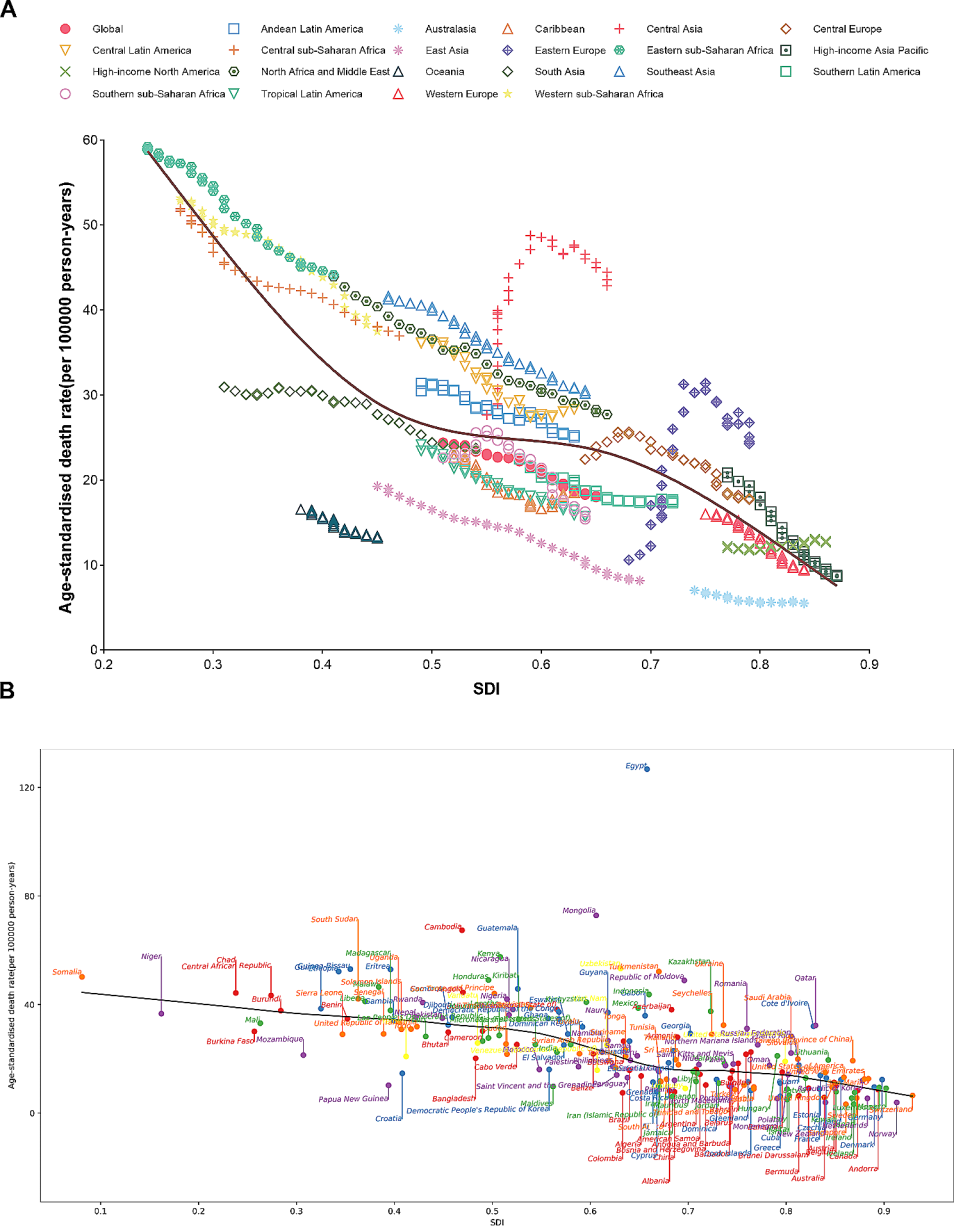
ASMR of liver cirrhosis and other chronic liver diseases by SDI: (**A**) ASMR in global and 21 GBD regions, 1990–2019. (**B**) ASMR in 204 countries and territories, 2019. Expected values based on sociodemographic index and disease rates in all locations are shown as the black line. ASMR, age-standardized mortality rate; GBD, global burden of diseases, injuries, and risk factors study; SDI, sociodemographic index

From 1990 to 2019, there were generally nonlinear relationships between the ASMR of liver cirrhosis and other chronic liver diseases and SDI at both global and regional levels. The expected values based on SDI and ASMR in all locations indicated a gradual decrease in ASMR with improvements in SDI, reflecting general expected trends (Fig. 6A). At the regional level, Central Latin America, Andean Latin America, Central Asia, Southeast Asia, and North Africa and the Middle East had a higher burden of liver cirrhosis and other chronic liver diseases compared to the expected levels based on SDIs from 1990 to 2019. Conversely, Oceania, East Asia, and Australasia exhibited a significantly lower burden than expected. In regions such as Central Europe, Eastern Europe, Central Asia, and Southern Sub-Saharan Africa, the burden of liver cirrhosis and other chronic liver diseases initially increased and then decreased with the enhancement of SDI over time. On a global scale, the observed burden of liver cirrhosis and other chronic liver diseases was lower than the expected level based on the SDIs from 1990 to 2019 (Fig. 6A).

At the national level, a nonlinear association was also observed between the ASMR and the SDI value. Countries like Egypt, Mongolia, Cambodia, Zambia, and several others had a higher ASMR than the expected value, while countries like Colombia, Norway, New Zealand, and several others had a lower ASMR than expected based on the SDIs (Fig. 6B). Nonlinear associations between the SDI and ASIR and ASDR of liver cirrhosis and other chronic liver diseases were also detected at the regional and national levels (Figure S20 and S21).

These findings highlight the complex relationships between the ASMR of liver cirrhosis and other chronic liver diseases, SDI, and geographical variations, indicating the need for further investigation into the underlying factors contributing to the observed burdens.

### Relative risk estimation of ASMR in males and females

The coefficients and overall intercepts of the various predictors were determined through using of GLMs (Table 2). The predictive formula for ASMR is:

**Table 2:** GLM results on RR of ASMR for cirrhosis and other chronic liver diseases

coefficients		value	*P* value
**α0**		36.663	< 0.001
**α1**			
	Gender = male	26.315	< 0.001
	Gender = female	0	
**α2**		-32.187	< 0.001
**α3**		1.725	0.007
**α4**		-2.299	0.006
**α5**			
	Gender = male	-18.273	0.041
	Gender = female	0	

Y = 36.663 + 26.315×Gender − 32.187×SDI + 1.725×AlcoholUse − 2.299×SDI&AlcoholUse − 18.273×Gender&SDI (Gender = male).

Y = 36.663–32.187×SDI + 1.725×AlcoholUse − 2.299×SDI&AlcoholUse (Gender = female).

RR for males relative to females in ASMR of liver cirrhosis and other chronic liver diseases was calculated as follows when both males and females without any alcohol consumption:

RR=(36.663 + 26.315–32.187×SDI-18.273)/(36.663–32.187×SDI)=(44.705–32.187×SDI)/(36.663–32.187×SDI)

SDI values exhibited variations across different countries and regions. When considering both males and females without any alcohol consumption, there was a correlation between the relative risk (RR) of death rates for males compared to females and SDI values. In 2019, the global mortality rate among males was 1.51 times higher than that among females (SDI in 2019 was known). This suggests a gender disparity in mortality rates, with males being more vulnerable to certain factors associated with liver cirrhosis and other chronic liver diseases compared to females.

## Discussion

As the major causes of liver cirrhosis and other chronic liver diseases is largely preventable and treatable, it is crucial for policymakers in national health-care systems to have a comprehensive understanding of the incidence, mortality and DALYs associated with each causal factor. This knowledge allows for the implementation of targeted interventions aimed at reducing premature deaths and morbidity related to liver cirrhosis and other chronic liver diseases. Therefore, there is a pressing need for a detailed assessment of the global and regional disease burden, particularly in terms of mortality, incidence, and DALYs attributable to liver cirrhosis and other chronic liver diseases. This study represents an important contribution as it is, to the best of our knowledge, the first comprehensive analysis showcasing the burden of liver cirrhosis and other chronic liver diseases worldwide between 1990 and 2019. It provides a detailed account of the numbers and age-standardized rates of mortality, incidence, and DALYs stratified by five etiologies globally, regionally, and nationally. Across the globe, there has been a steady increase in numbers of mortality, incidence, and DALYs associated with liver cirrhosis and other chronic liver diseases over this period, likely due to population growth and aging. These factors have contributed to a relatively inadequate provision of health care resources, particularly in underdeveloped regions and countries. However, it is worth noting that age-standardized rates of mortality and DALYs have shown a declining trend from 1990 to 2019, indicating some improvements in addressing the disease burden of liver cirrhosis and other chronic liver diseases. Additionally, decreases in ASMR and ASDR suggest potential future improvements in the overall situation. Although the ASIR has remained relatively stable, the declines observed in mortality and DALYs may be associated with increased long-term survival of patients.

The age-standardized rates of liver cirrhosis and other chronic liver diseases varied across the five etiologies studied. It is evident that hepatitis B virus, hepatitis C virus, and alcohol use were still the primary causes of liver cirrhosis and other chronic liver diseases deaths at the global level. In 2019, these three factors accounted for 74.61% of liver cirrhosis and other chronic liver diseases-related mortalities. Notably, the prevalence trends indicated a more significant decline in HBV compared to other factors, with a gradual decrease in the proportion of mortalities attributed to HBV since 2009. This decline can be attributed, in some part, to routine immunization with the hepatitis B vaccine in many regions [19]. Furthermore, the Western Sub-Saharan Africa region exhibited the highest ASMR for liver cirrhosis and other chronic liver diseases caused by HBV in 2019. This can be attributed to various factors such as high temperatures, a high rate of invisible HBV infection, and inadequate interruption of mother-to-child transmission. These conditions make the population more susceptible to HBV infection, leading to a silent epidemic of the virus in this region [20–22]. Implementing routine immunization programs targeting HBV transmission and improving healthcare infrastructure have the potential to effectively control the incidence of hepatitis B-related liver cirrhosis and other chronic liver diseases deaths in African and other Asian countries.

Previous studies have indicated that hepatitis C virus is the leading cause of liver cirrhosis and other chronic liver diseases in developed countries and regions [1, 3, 23]. However, our study reveals that HCV remains the predominant etiology not only in high-income regions such as Asia Pacific and North America but also in less developed areas including North Africa, the Middle East, and Southern Latin America. The dominance of HCV as a significant risk factor for liver cirrhosis and other chronic liver diseases in various economic settings highlights the importance of addressing this viral infection on a global scale. While prevention and control efforts have made substantial progress in high-income regions, there is a pressing need to extend these measures to less developed areas where HCV remains a significant public health concern. It is essential to establish robust surveillance networks, strengthen laboratory capacities, and promote information sharing to facilitate a comprehensive understanding of the burden of HCV-related liver cirrhosis and other chronic liver diseases across different regions. Luckily, significant progress has been achieved in the field of hepatitis C virus treatment over the past few decades, culminating in HCV becoming the first chronic viral infection that can be cured [24]. This remarkable development can be attributed to the introduction of direct-acting antiviral agents (DAAs), which have revolutionized antiviral therapy and cure rates exceeding 98% across various patient populations infected with HCV [24]. These medications directly target specific viral proteins essential for HCV replication, resulting in rapid suppression of viral replication and ultimately achieving sustained virological response (SVR), which is defined as undetectable levels of HCV RNA in the blood 12 weeks after completion of treatment and serves as a marker for viral eradication. The availability of highly effective and well-tolerated DAA regimens has transformed HCV management, offering the potential to achieve viral eradication and improve patient outcomes.

Noteworthy, we observed that alcohol use is a significant contributor to liver cirrhosis and other chronic liver diseases, as supported by our findings and previous evidence [10]. Specifically, Mongolia and Moldova emerged as the countries with the highest age-standardized rates of liver cirrhosis and other chronic liver diseases attributed to alcohol use. Excessive alcohol consumption in Mongolia can be attributed to various factors, including geographical location, cultural influences from neighboring Russia, and the economic dependence on the alcohol industry [25, 26]. It is evident from our study and previous research that there is a pressing need for a comprehensive approach to reduce alcohol consumption in these countries in order to alleviate the burden of liver cirrhosis and other chronic liver diseases [3, 10, 27].

Furthermore, non-alcoholic fatty liver disease and obesity have been identified as significant risk factors for liver cirrhosis and other chronic liver diseases [11, 12]. Our study revealed a global increase in the incident cases of NAFLD-associated liver cirrhosis and other chronic liver diseases from 1990 to 2019. This rising trend was observed not only in economically underdeveloped countries like Kazakhstan and Mongolia but also in developed nations such as the United Kingdom and Finland. The growing prevalence of obesity and metabolic syndrome is closely associated with the increasing incidence of NAFLD. Therefore, it is reasonable to expect a surge in the morbidity rates associated with this condition. Given the lack of effective preventive or therapeutic interventions for NAFLD, maintaining a healthy diet and engaging in regular exercise become particularly important strategies [13].

Factors such as smoking and aflatoxin B1 were categorized as “other causes” in the Global Burden of Disease (GBD) 2019 study, along with all risk factors not mentioned earlier [14]. Analyzing the statistical results of age-standardized mortality rates (ASMR), age-standardized death rates (ASDR), and age-standardized incidence rates (ASIR) for liver cirrhosis and other chronic liver diseases from other causes, we discovered that the disease burden associated with these causes was more severe than anticipated, surpassing that caused by non-alcoholic fatty liver disease (NAFLD) over the past three decades. Hence, there is a need for greater implementation of prevention measures to reduce exposure to aflatoxin B1 and smoking.

On a global scale, liver cirrhosis and other chronic liver diseases exhibits a significant sex-based disparity, with the mortality rate in males being 1.51 times higher than in females in 2019 [6, 28]. This discrepancy between males and females can be attributed to factors such as sex hormones, sex chromosomes, immunity, and behaviors (particularly alcohol use and smoking) [29, 30]. Therefore, it is highly recommended to adopt sex-specific prevention and treatment strategies for liver cirrhosis and other chronic liver diseases [31].

Furthermore, we investigated the nonlinear associations between mortality, incidence, DALYs of liver cirrhosis and other chronic liver diseases with the SDI values of different regions and countries. In general, higher SDI values were correlated with a lower disease burden of liver cirrhosis and other chronic liver diseases from 1990 to 2019 across most of the 21 GBD regions [4, 32]. These findings are expected, as nations with higher income tend to exhibit lower mortality rates related to liver cirrhosis and other chronic liver diseases due to improved healthcare access and strengthened health infrastructure. However, certain regions and countries with higher SDI values still experienced a relatively high burden of liver cirrhosis and other chronic liver diseases, indicating that the disease burden cannot be solely inferred from SDI values. Overall, it is undeniable that better economic development in regions and countries can contribute to mitigating the disease burden associated with liver cirrhosis and other chronic liver diseases.

There are several limitations to consider in this study. Firstly, since this study relied on secondary analysis of data from the Global Burden of Disease 2019, the accuracy of the conclusions is dependent on the quality of the data collected by GBD and the research methods employed in this analysis. Secondly, the classification of risk factors for liver cirrhosis and other chronic liver diseases in GBD 2019 was limited to five etiologies, which means that only a subset of etiologies could be analyzed for their impact on disease burden. Further research is necessary to differentiate the specific factors included under the category of ‘other causes’ in this analysis, including autoimmune hepatitis as a potential sixth factor leading to liver cirrhosis and other chronic liver diseases.

## Conclusion

In summary, this study confirms that liver cirrhosis and other chronic liver diseases is a significant global health issue, necessitating the attention of primary care physicians, specialists, and health policymakers to implement appropriate preventive measures and enhance awareness regarding early detection, diagnosis, and treatment. From 1990 to 2019, the global ASMR and ASDR for the five specified causes of liver cirrhosis and other chronic liver diseases demonstrated a decline. However, among the five ASIRs, only the incidence rate for hepatitis B showed a decreasing trend. Given that liver cirrhosis and other chronic liver diseases can be prevented and its progression slowed through appropriate interventions, variations among risk factors, age and gender groups, as well as different regions and countries should be taken into account in disease control, prevention, and treatment strategies for liver cirrhosis and other chronic liver diseases.

### Electronic supplementary material

Below is the link to the electronic supplementary material.

Supplementary Material 1

## Data Availability

The datasets analyzed during the current study are publicly available from the Global Health Data Exchange query tool (https://ghdx.healthdata.org/gbd-results-tool). The datasets supporting the conclusions of this article are available from the corresponding author upon reasonable request.
