# Supplementary material for: Global burden of liver cirrhosis and other chronic liver diseases caused by specific etiologies from 1990 to 2019
Source: BMC Public Health. 2024 Feb 3;24:363. doi: 10.1186/s12889-024-17948-6 (PMC10837876; doi:10.1186/s12889-024-17948-6)
Supplement: Supplementary file 1 — Supplementary Material 1 [file 12889_2024_17948_MOESM1_ESM.docx]

**
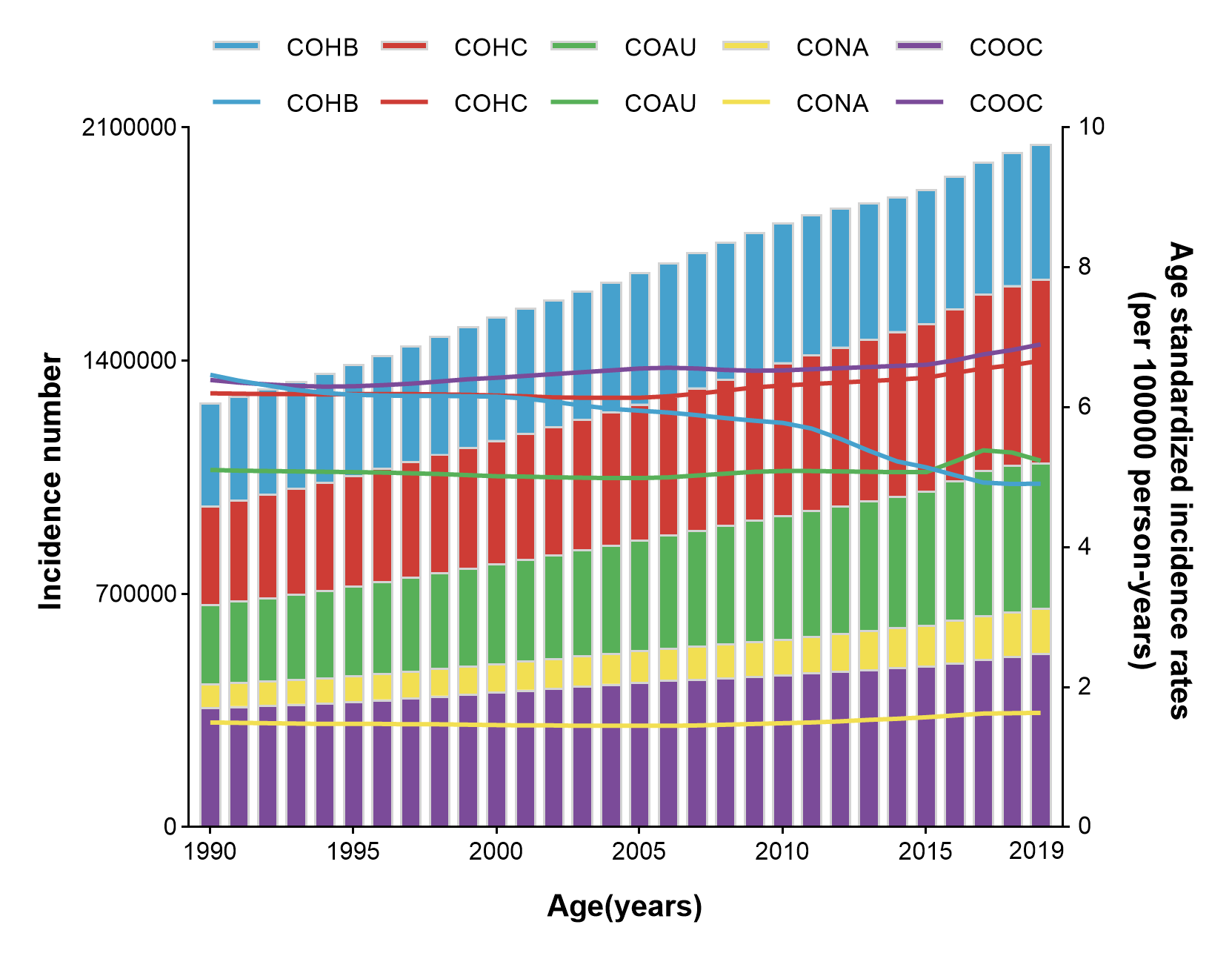
**

Fig S1: Number of incident cases and age-standardized incidence rates at the global level by etiology of liver cirrhosis and other chronic liver diseases, 1990–2019.


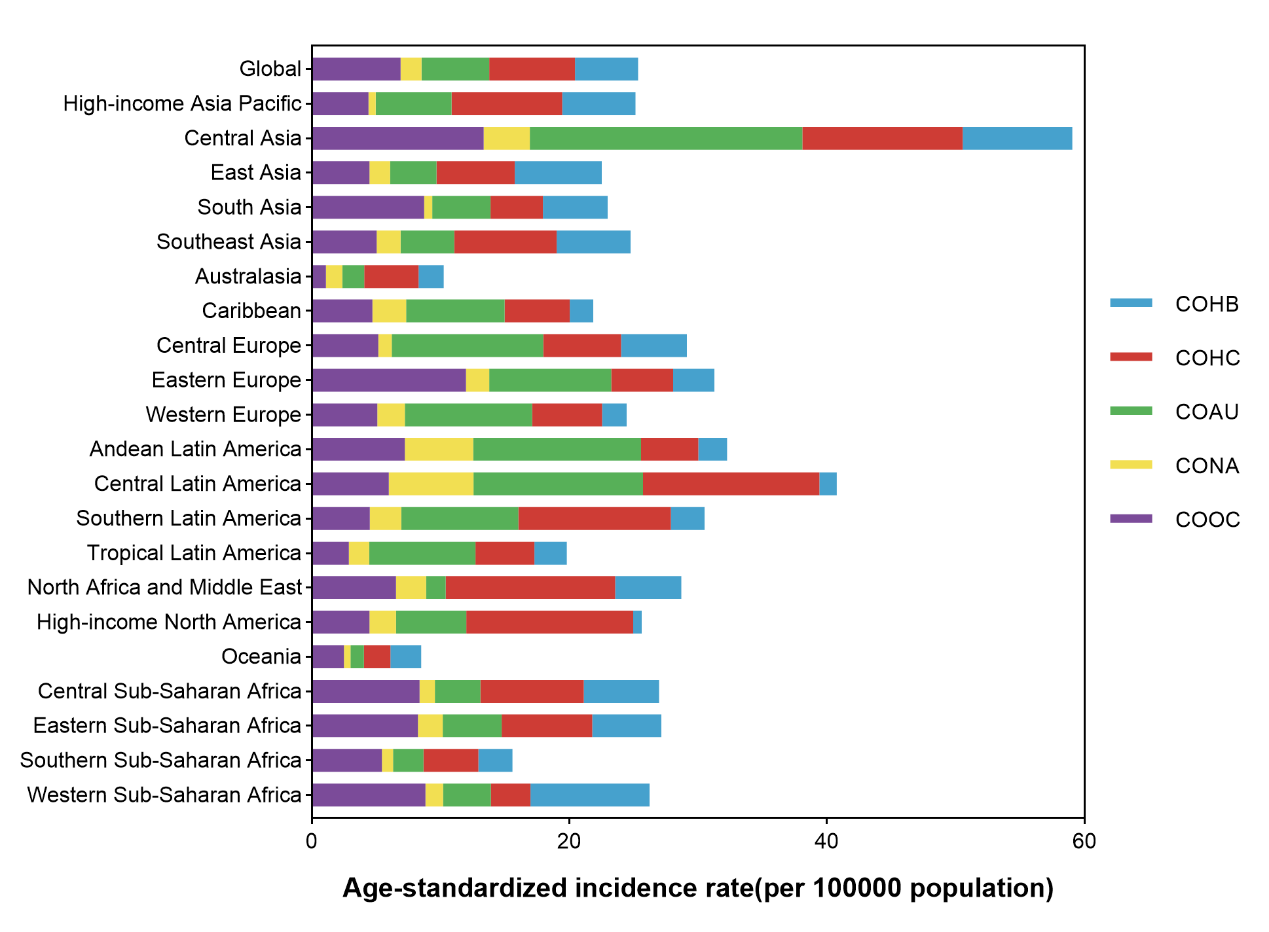


Fig S2: Age-standardized incidence rate for liver cirrhosis and other chronic liver diseases, by region and etiology, 2019.

**
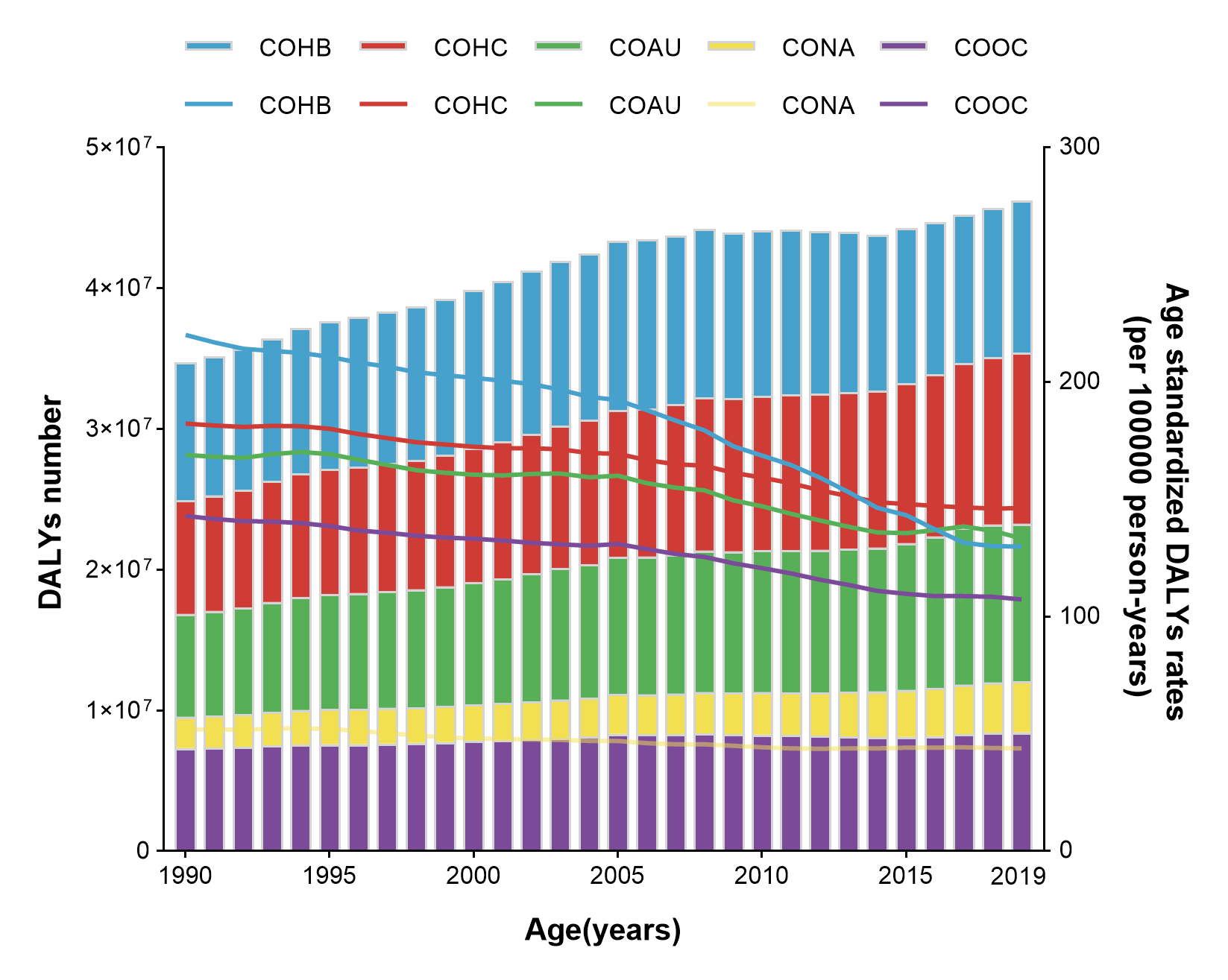
**

Fig S3: Number of DALYs and age-standardized DALYs rates at the global level by etiology of liver cirrhosis and other chronic liver diseases, 1990–2019.


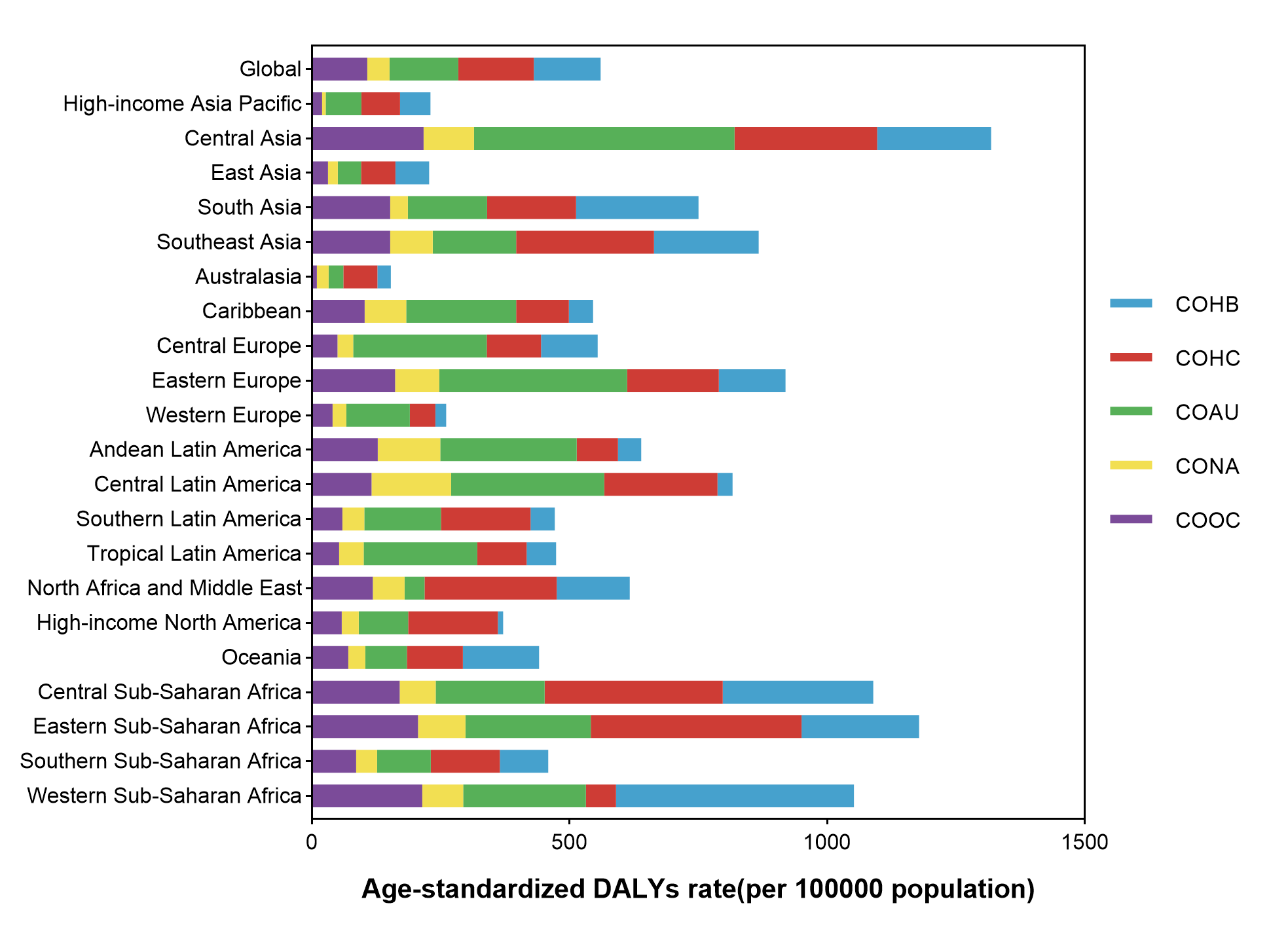


Fig S4: Age-standardized DALYs rate for liver cirrhosis and other chronic liver diseases, by region and etiology, 2019.


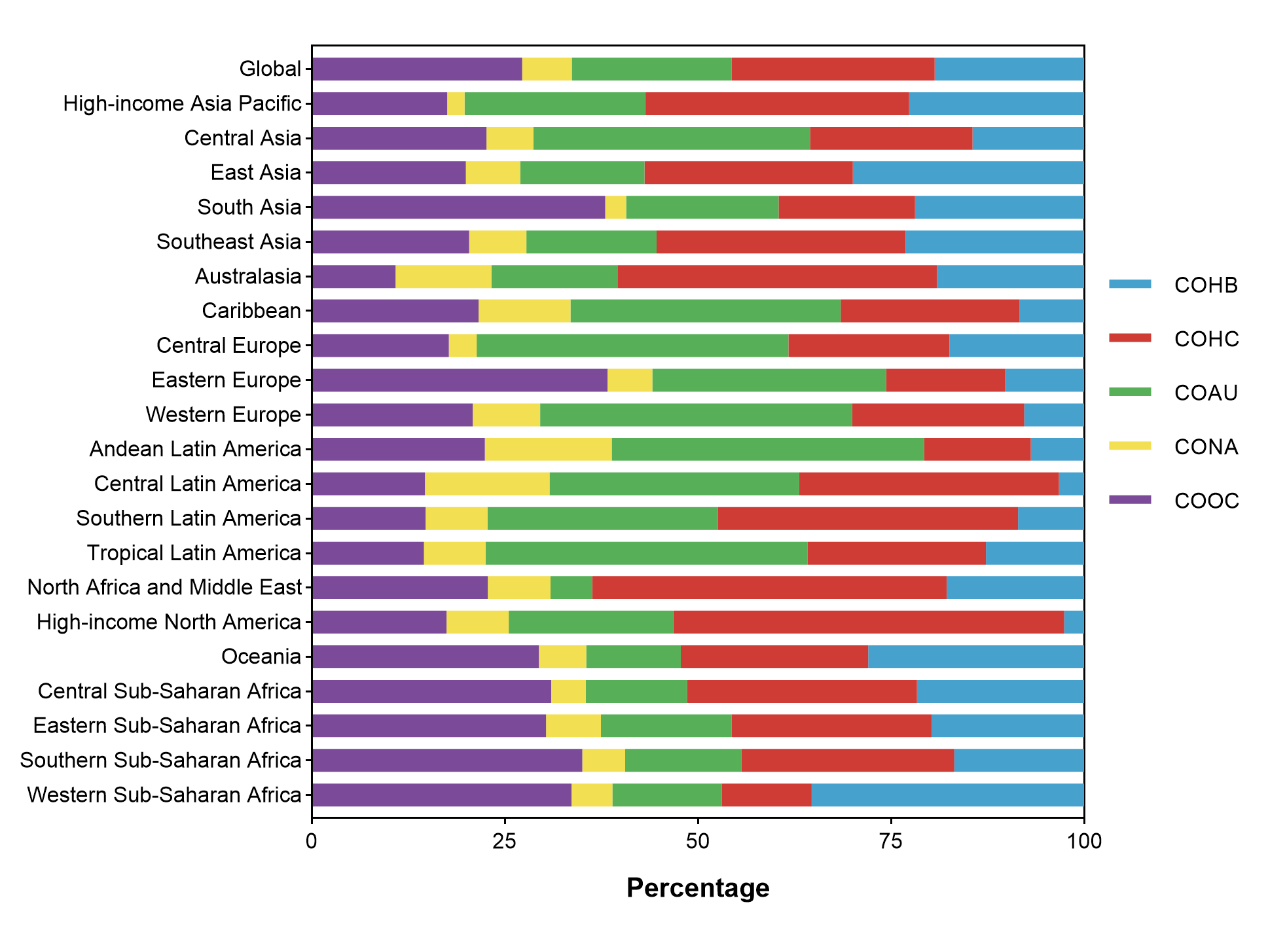


Fig S5: Contribution of COHB, COHC, COAU, CONA, and COOC to liver cirrhosis and other chronic liver diseases incident cases, both sexes, globally and by region, 2019


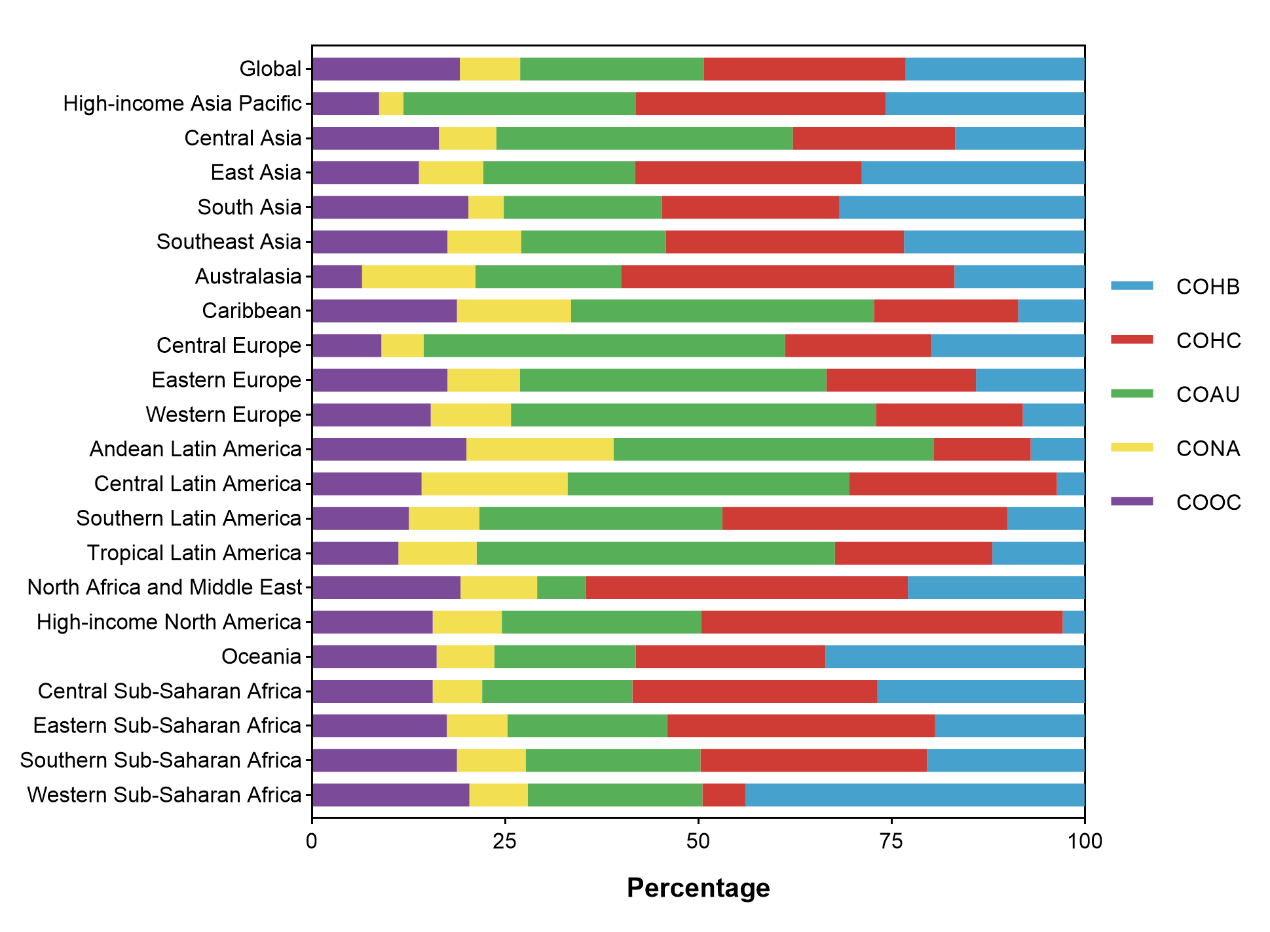


Fig S6: Contribution of COHB, COHC, COAU, CONA, and COOC to liver cirrhosis and other chronic liver diseases DALYs, both sexes, globally and by region, 2019


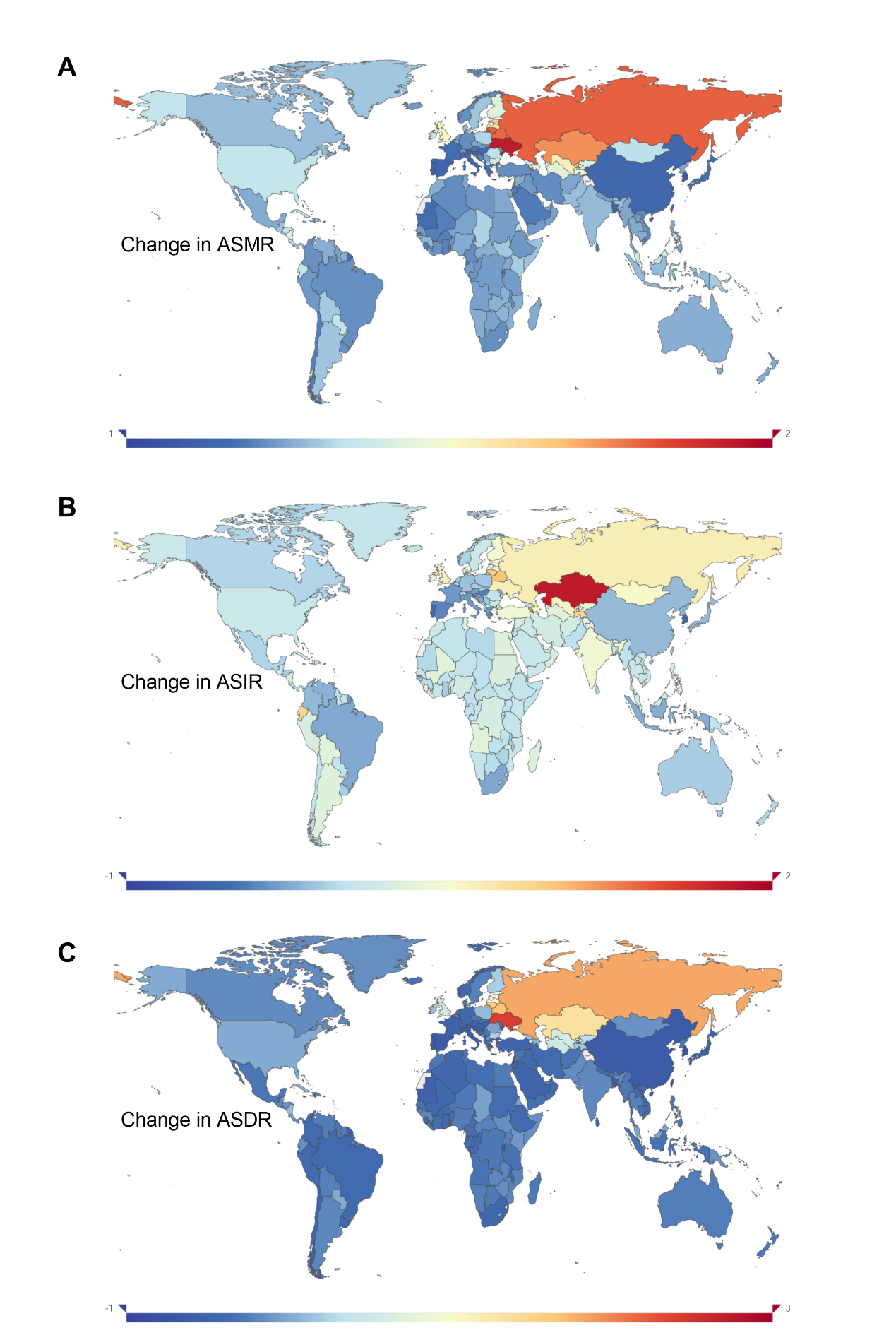


Fig.S7 The estimated percentage change of liver cirrhosis and other chronic liver diseases from 1990 to 2019: (A) The percentage change in ASMR. (B) The percentage change in ASIR. (C) The percentage change in ASDR.


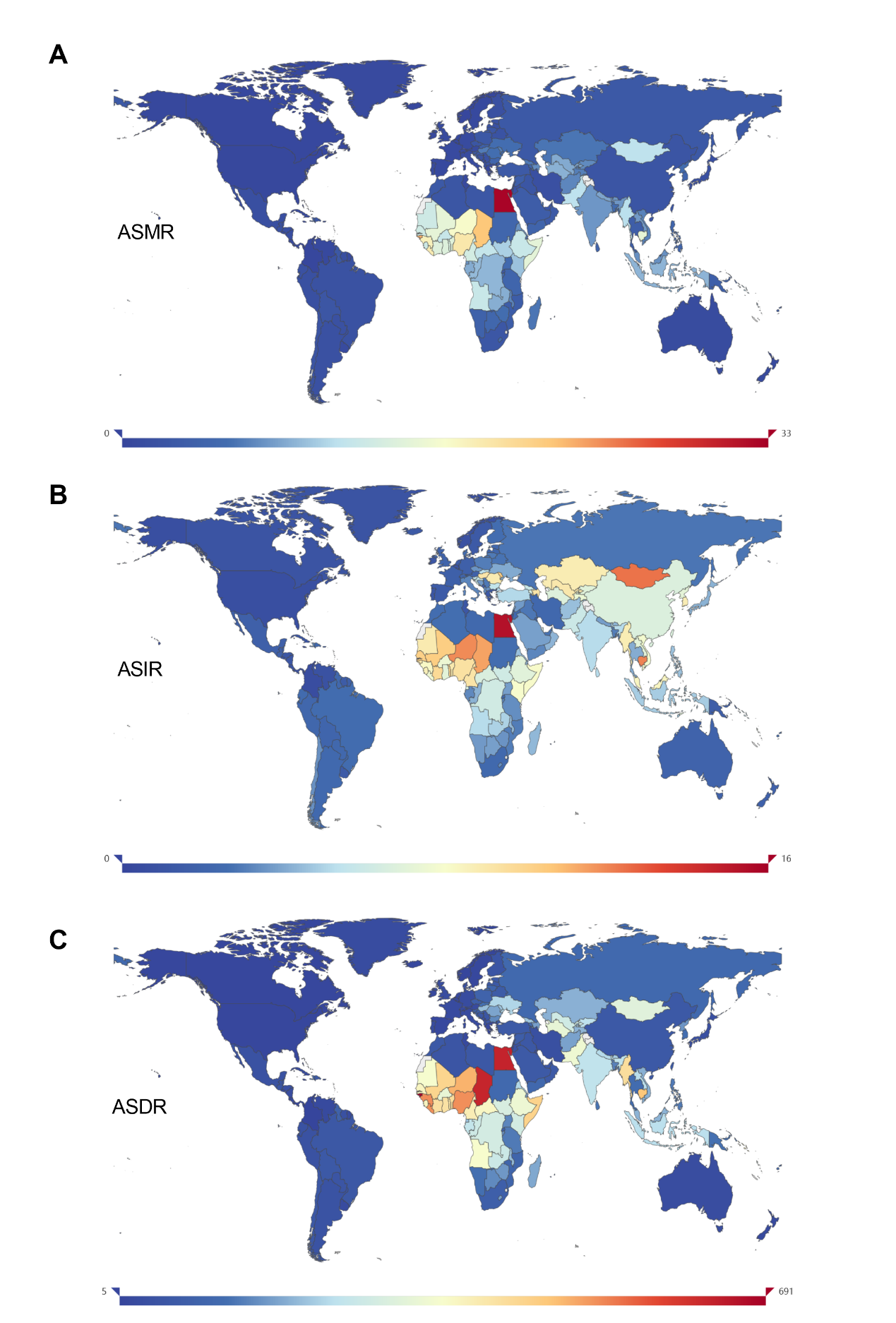


Fig.S8 The global age-standardized rate of COHB per 100 000 populations in 2019, by country and territory. (A) ASMR in 2019; (B) ASIR in 2019; (C) ASDR in 2019.


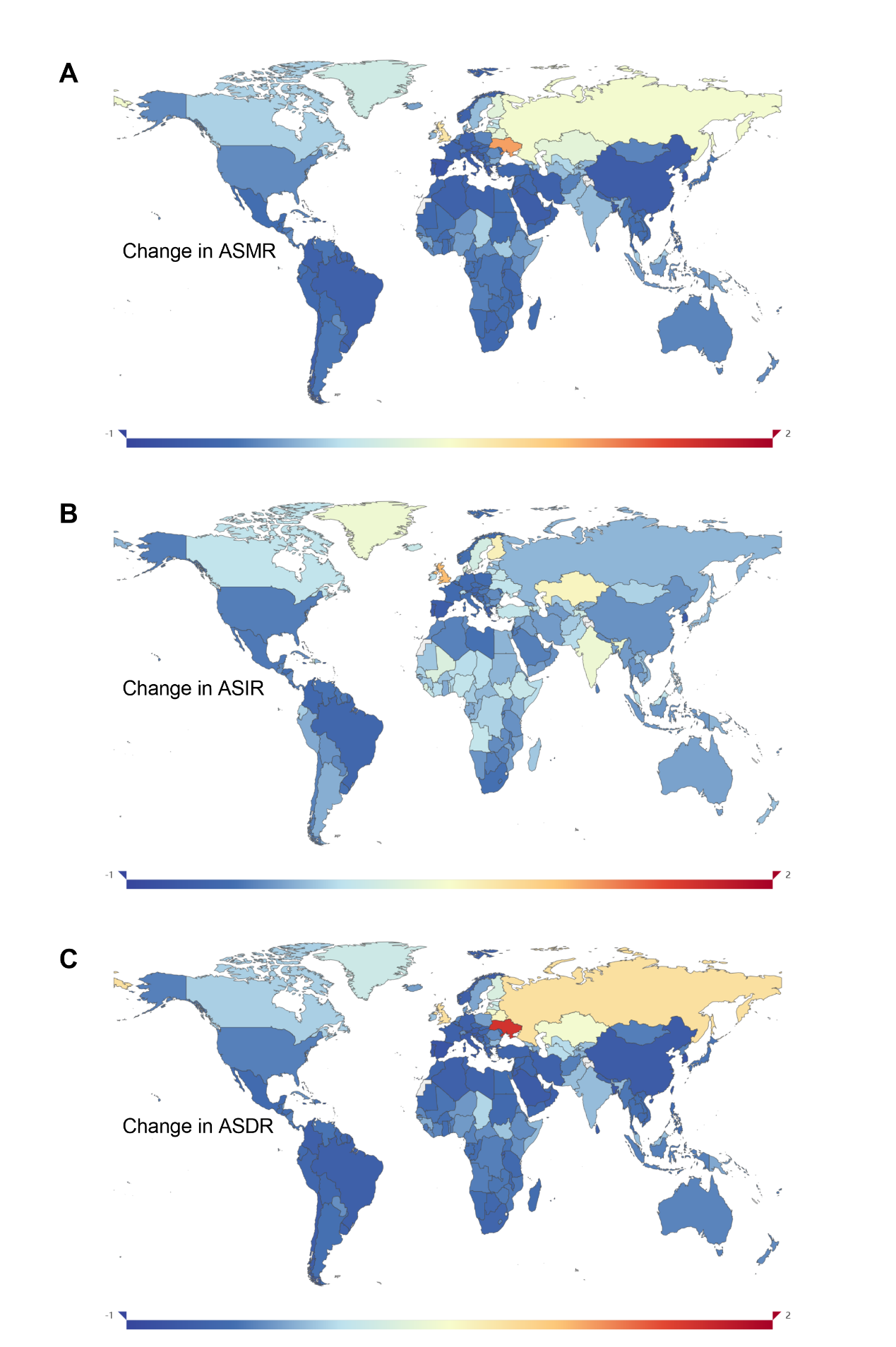


Fig.S9 The estimated percentage change of COHB from 1990 to 2019: (A) The percentage change in ASMR. (B) The percentage change in ASIR. (C) The percentage change in ASDR.


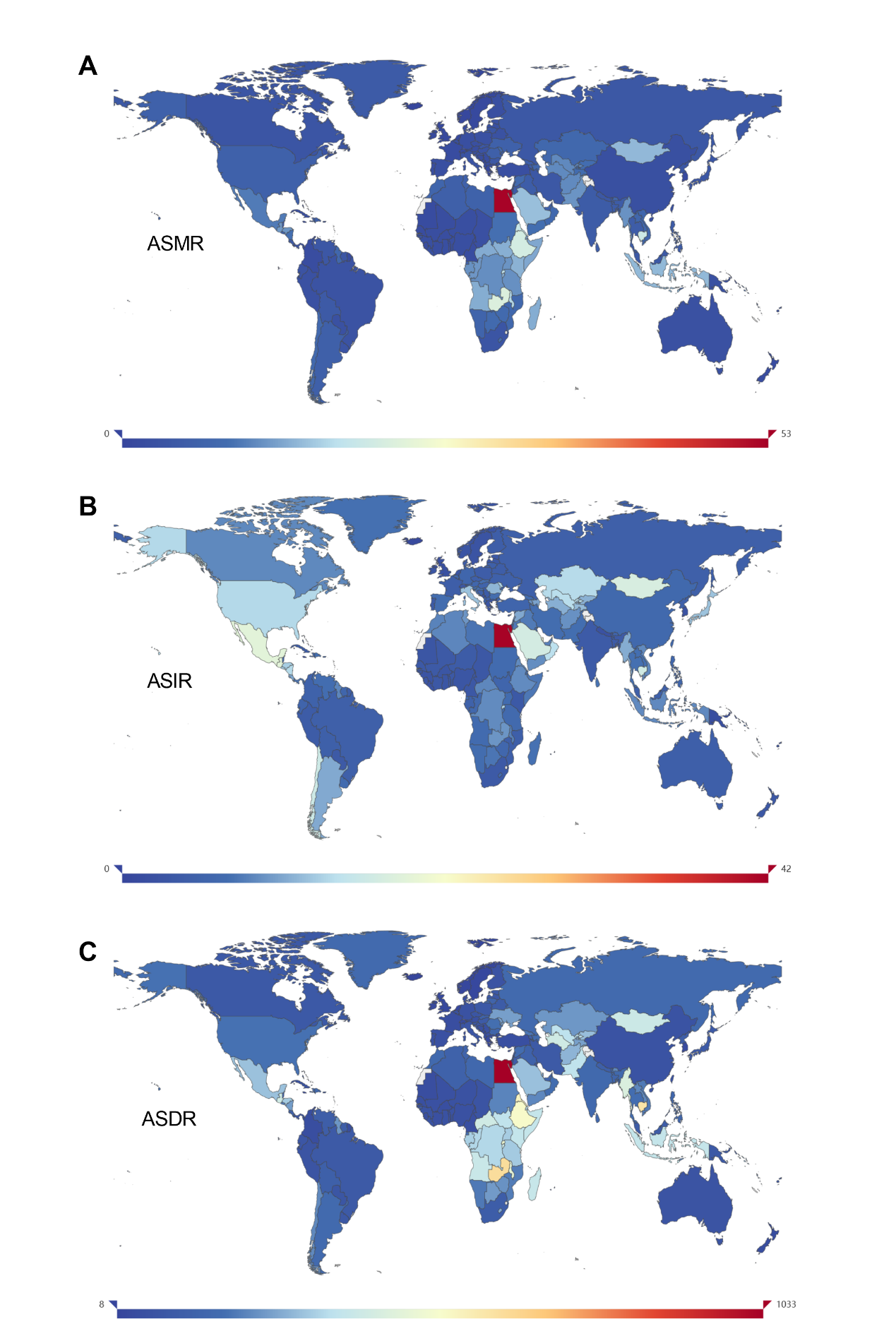


Fig.S10 The global age-standardized rate of COHC per 100 000 populations in 2019, by country and territory. (A) ASMR in 2019; (B) ASIR in 2019; (C) ASDR in 2019.


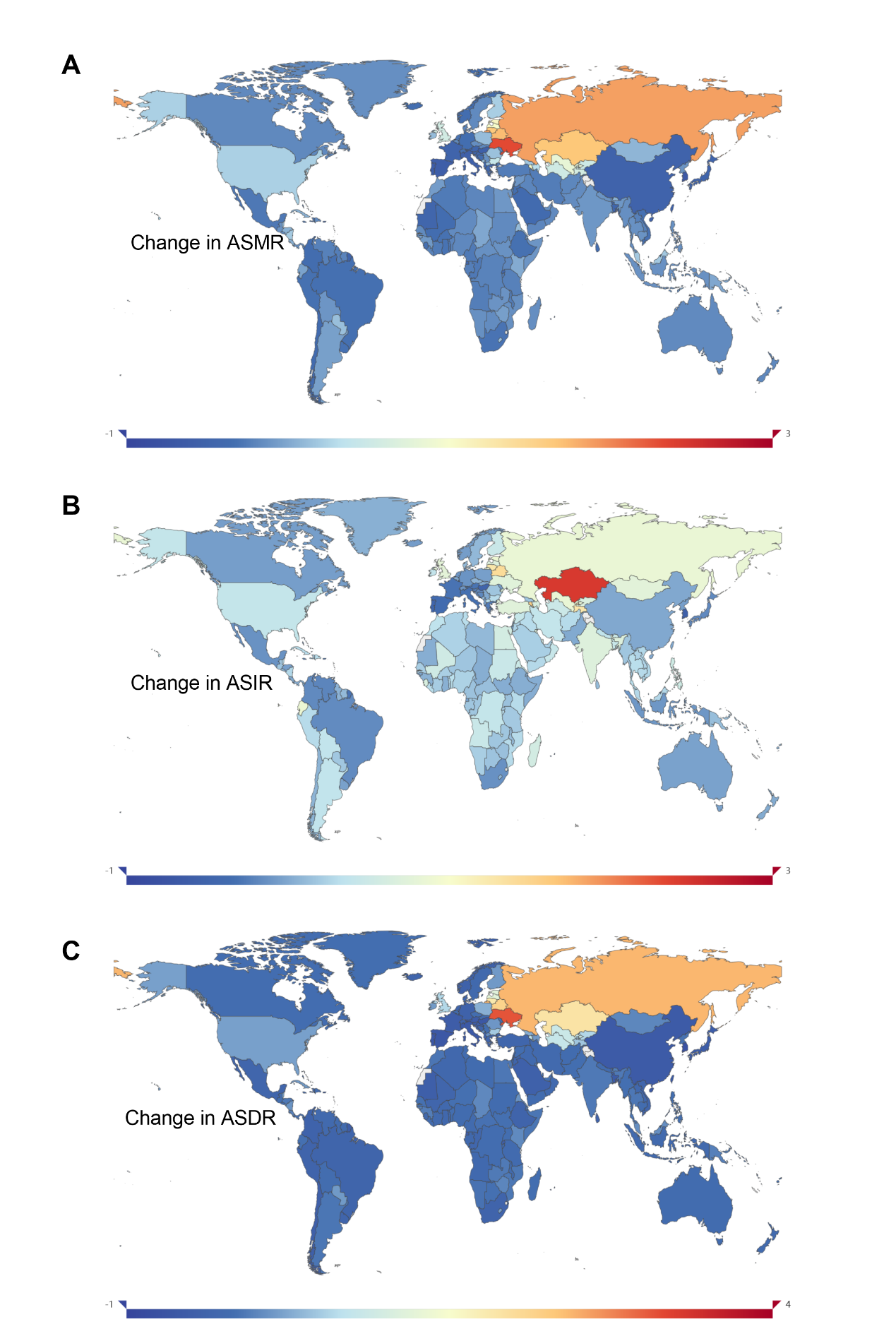


Fig.S11 The estimated percentage change of COHC from 1990 to 2019: (A) The percentage change in ASMR. (B) The percentage change in ASIR. (C) The percentage change in ASDR.


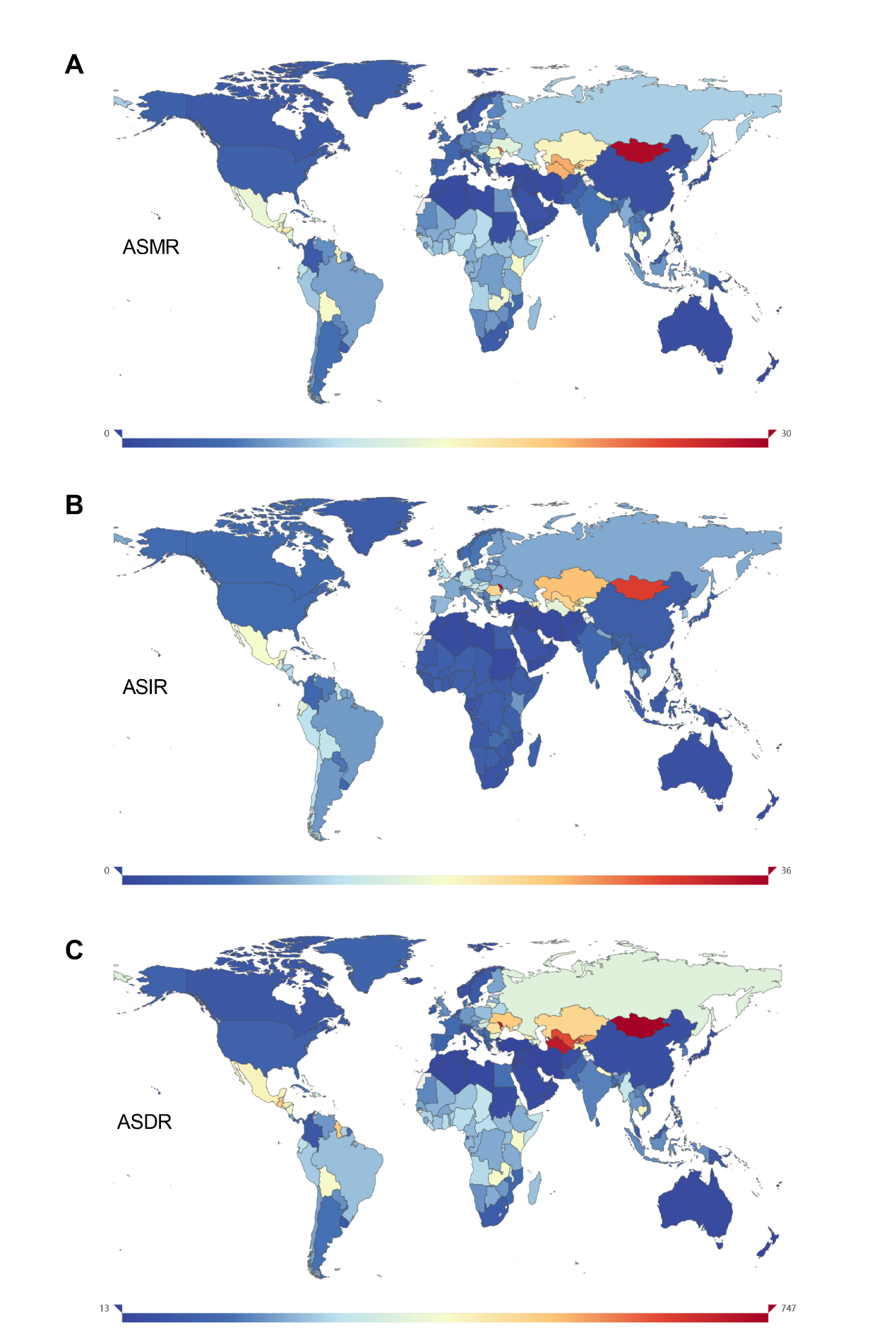


Fig.S12 The global age-standardized rate of COAU per 100 000 populations in 2019, by country and territory. (A) ASMR in 2019; (B) ASIR in 2019; (C) ASDR in 2019.


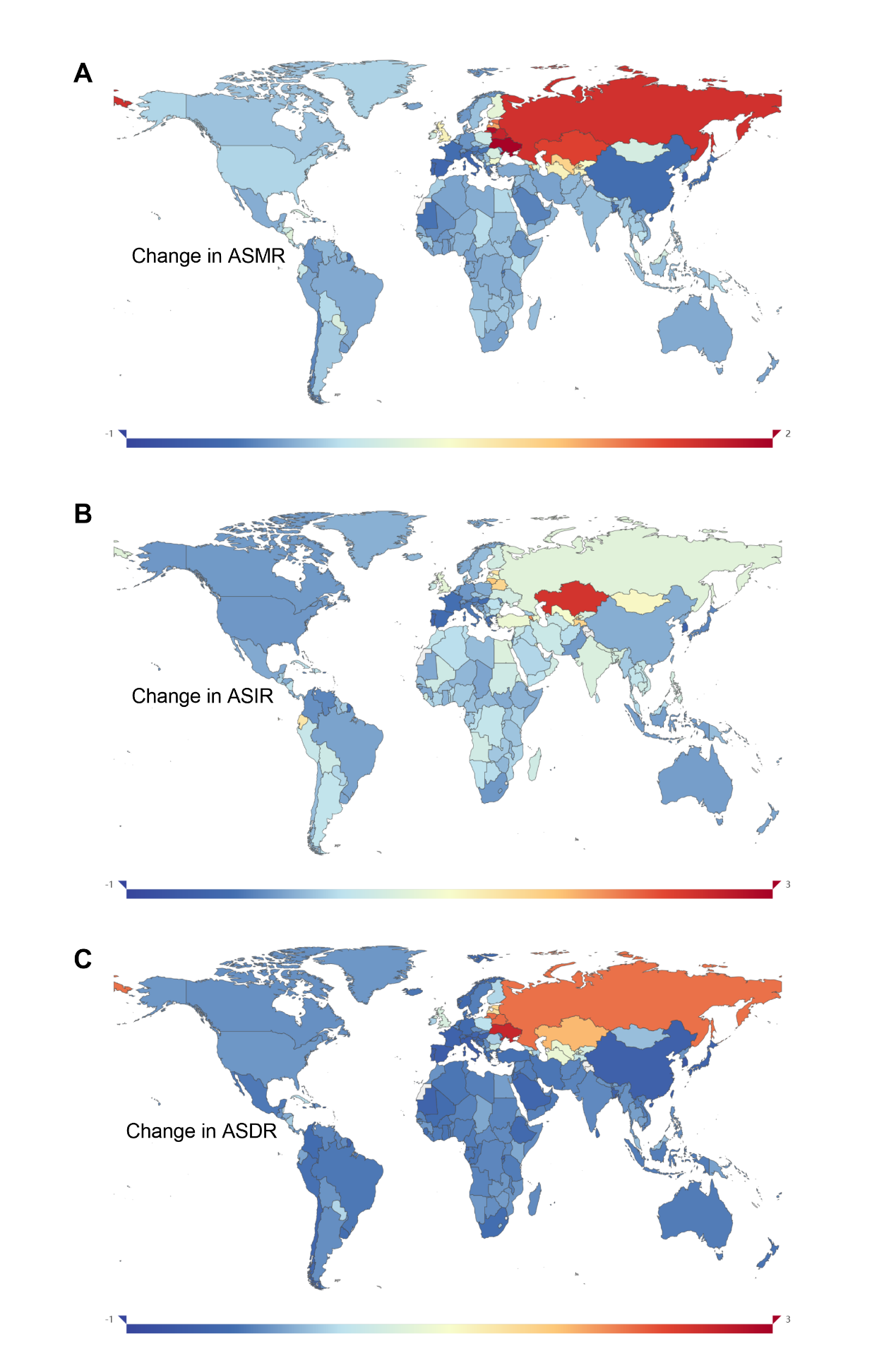


Fig.S13 The estimated percentage change of COAU from 1990 to 2019: (A) The percentage change in ASMR. (B) The percentage change in ASIR. (C) The percentage change in ASDR.


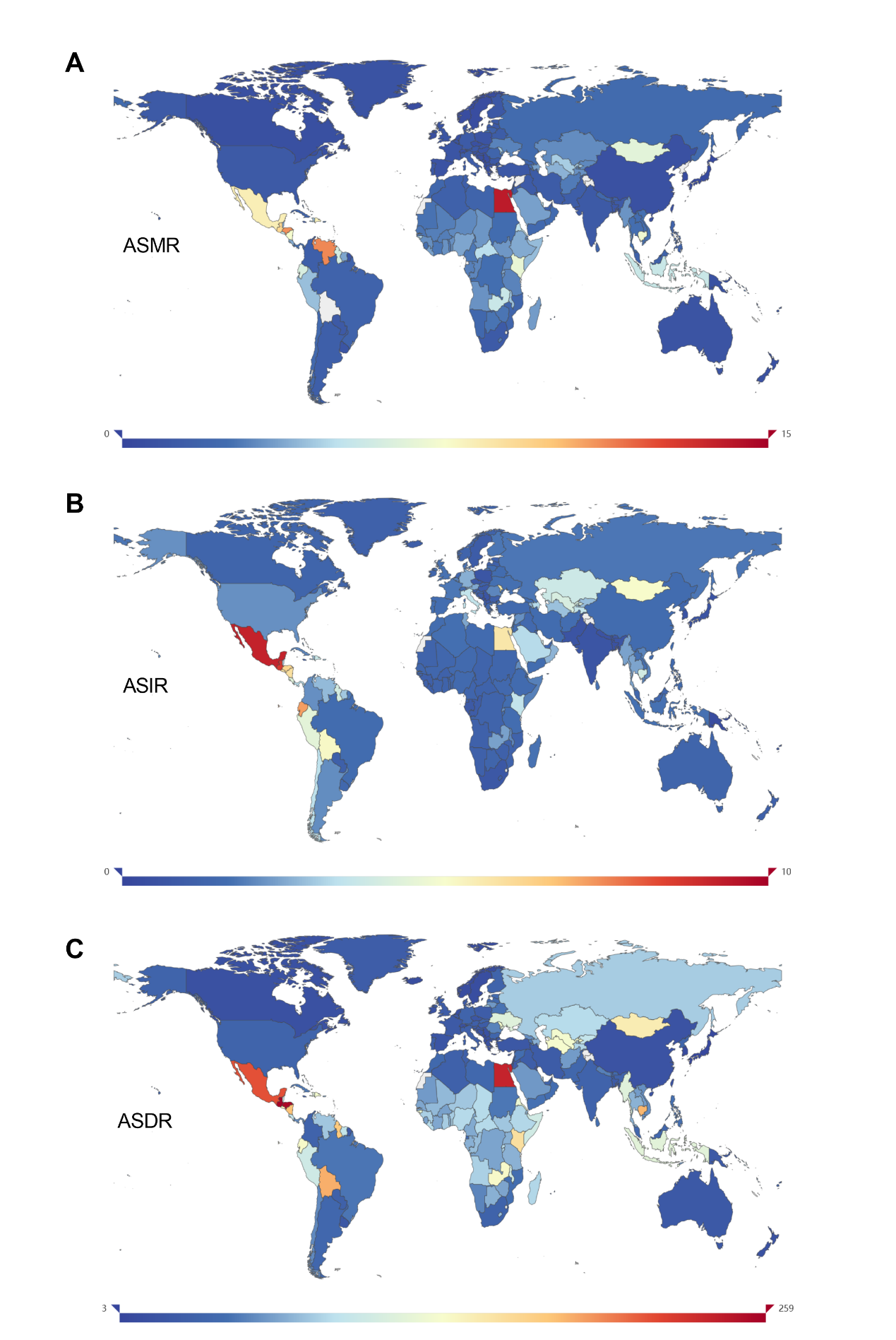


Fig. S14 The global age-standardized rate of CONA per 100 000 populations in 2019, by country and territory. (A) ASMR in 2019; (B) ASIR in 2019; (C) ASDR in 2019.


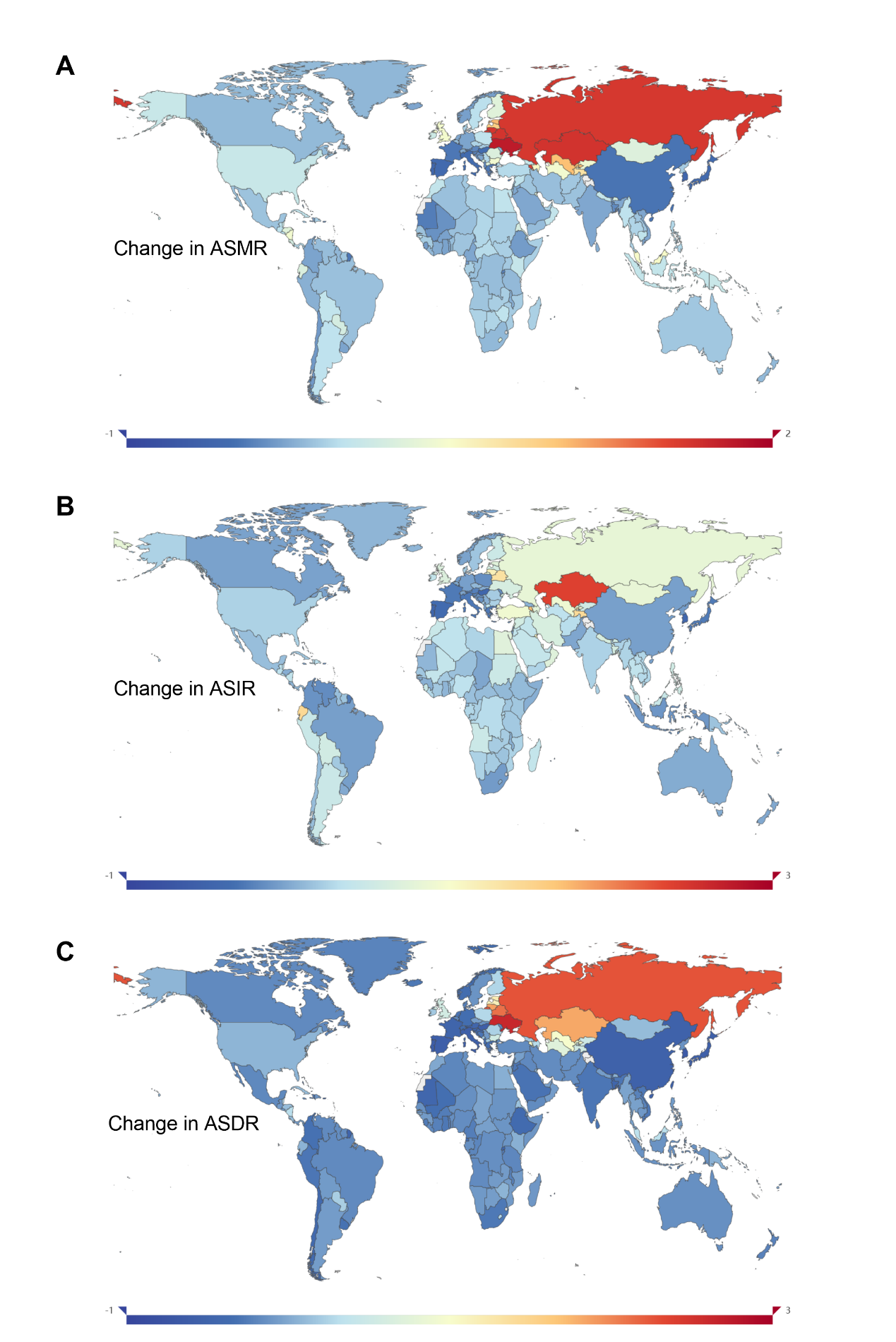


Fig.S15 The estimated percentage change of CONA from 1990 to 2019: (A) The percentage change in ASMR. (B) The percentage change in ASIR. (C) The percentage change in ASDR.


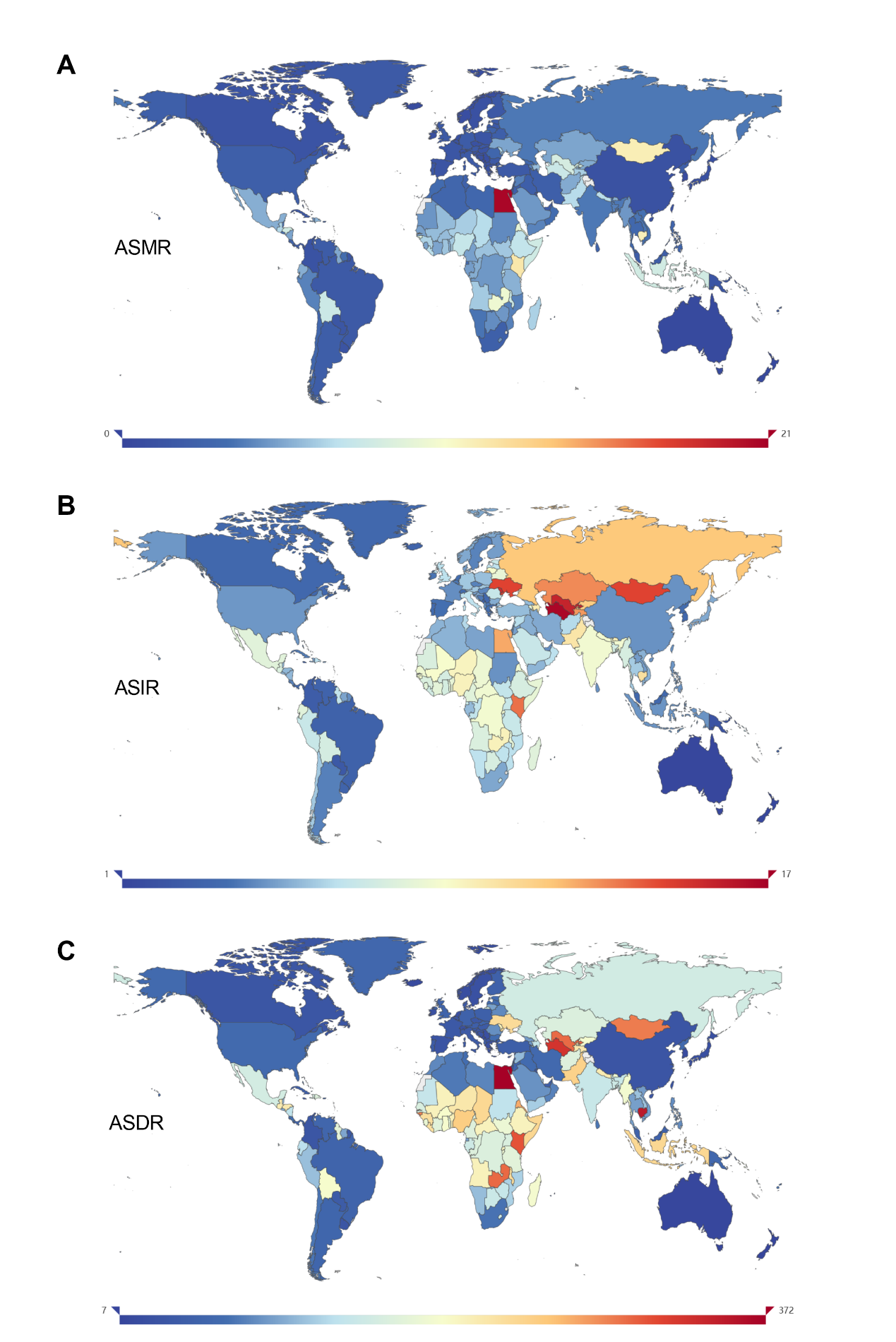


Fig.S16 The global age-standardized rate of COOC per 100 000 populations in 2019, by country and territory. (A) ASMR in 2019; (B) ASIR in 2019; (C) ASDR in 2019.


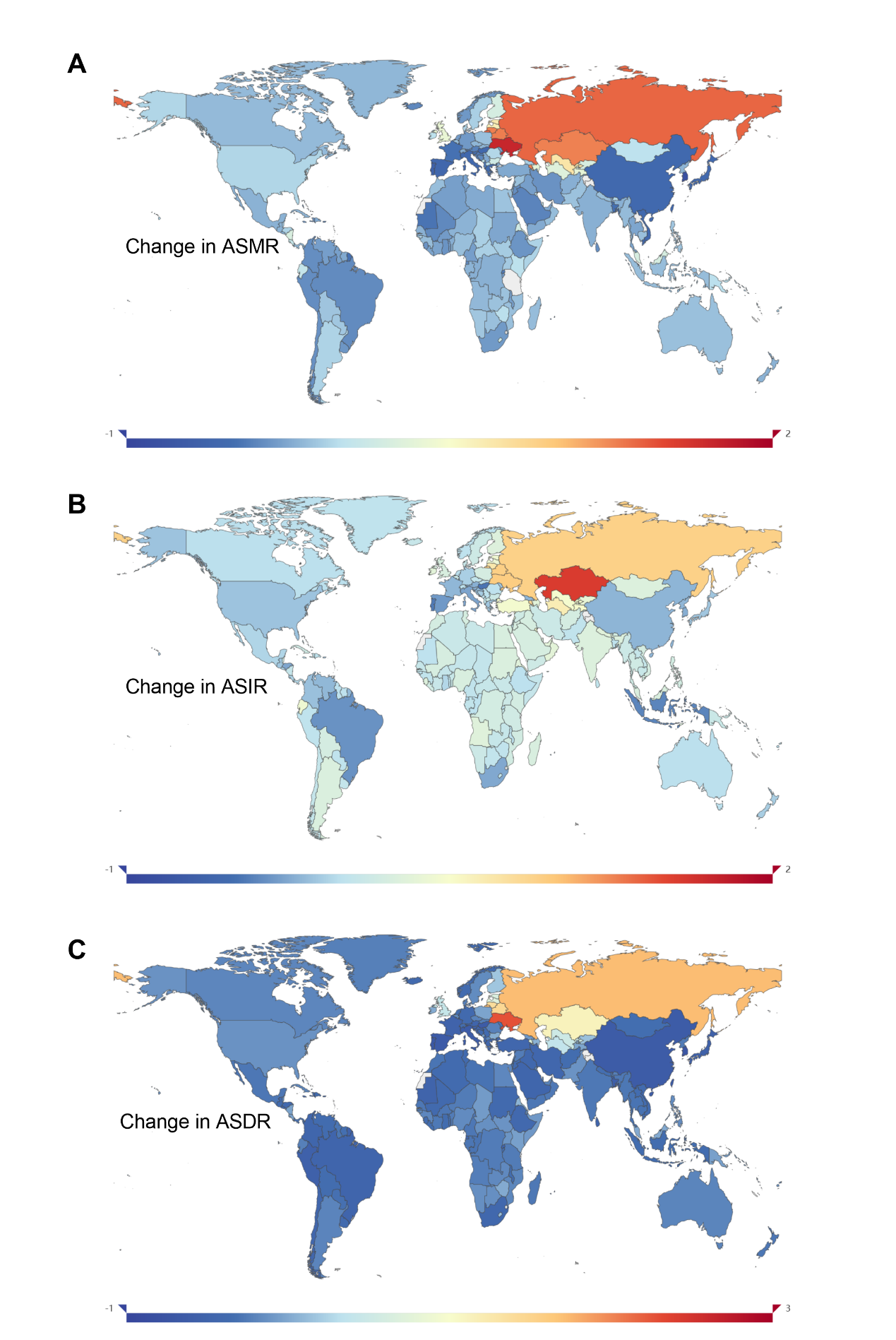


Fig.S17 The estimated percentage change of COOC from 1990 to 2019: (A) The percentage change in ASMR. (B) The percentage change in ASIR. (C) The percentage change in ASDR.


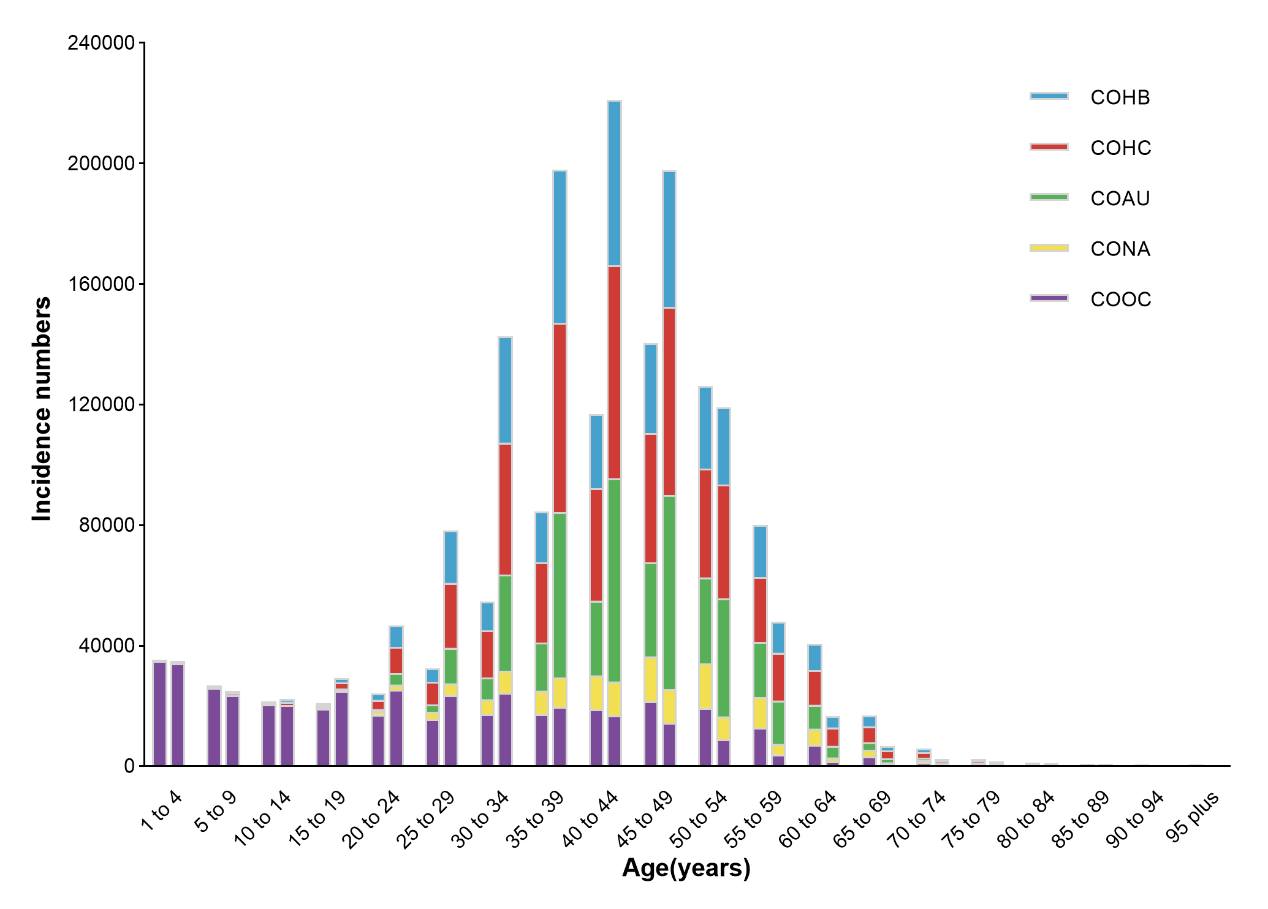


Fig.S18 Global liver cirrhosis and other chronic liver diseases incident cases by etiology and age for females and males, 2019. For each group, the left column showed case data in female and the right column shows data in male.


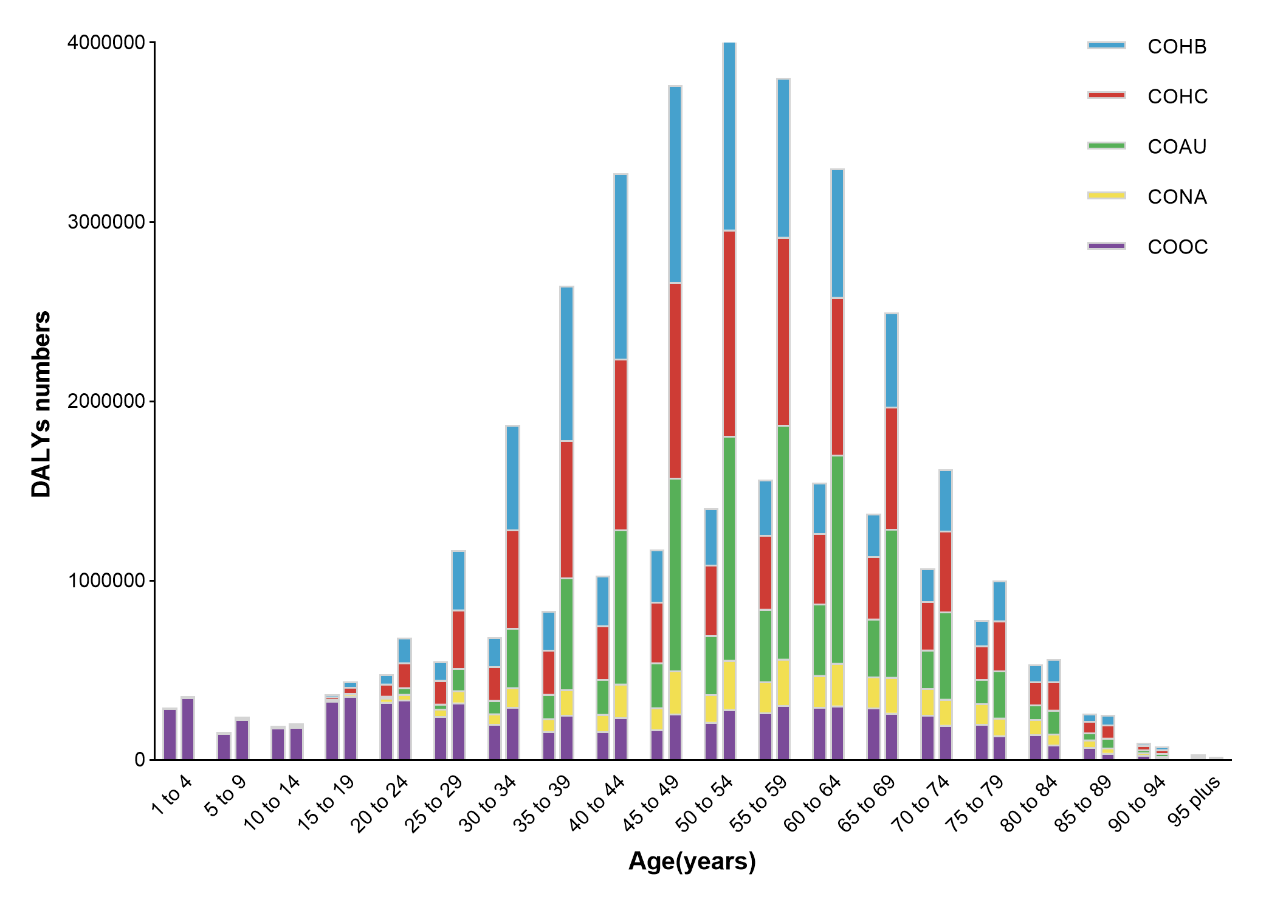


Fig.S19 Global liver cirrhosis and other chronic liver diseases DALYs by etiology and age for females and males, 2019. For each group, the left column showed case data in female and the right column shows data in male.

**
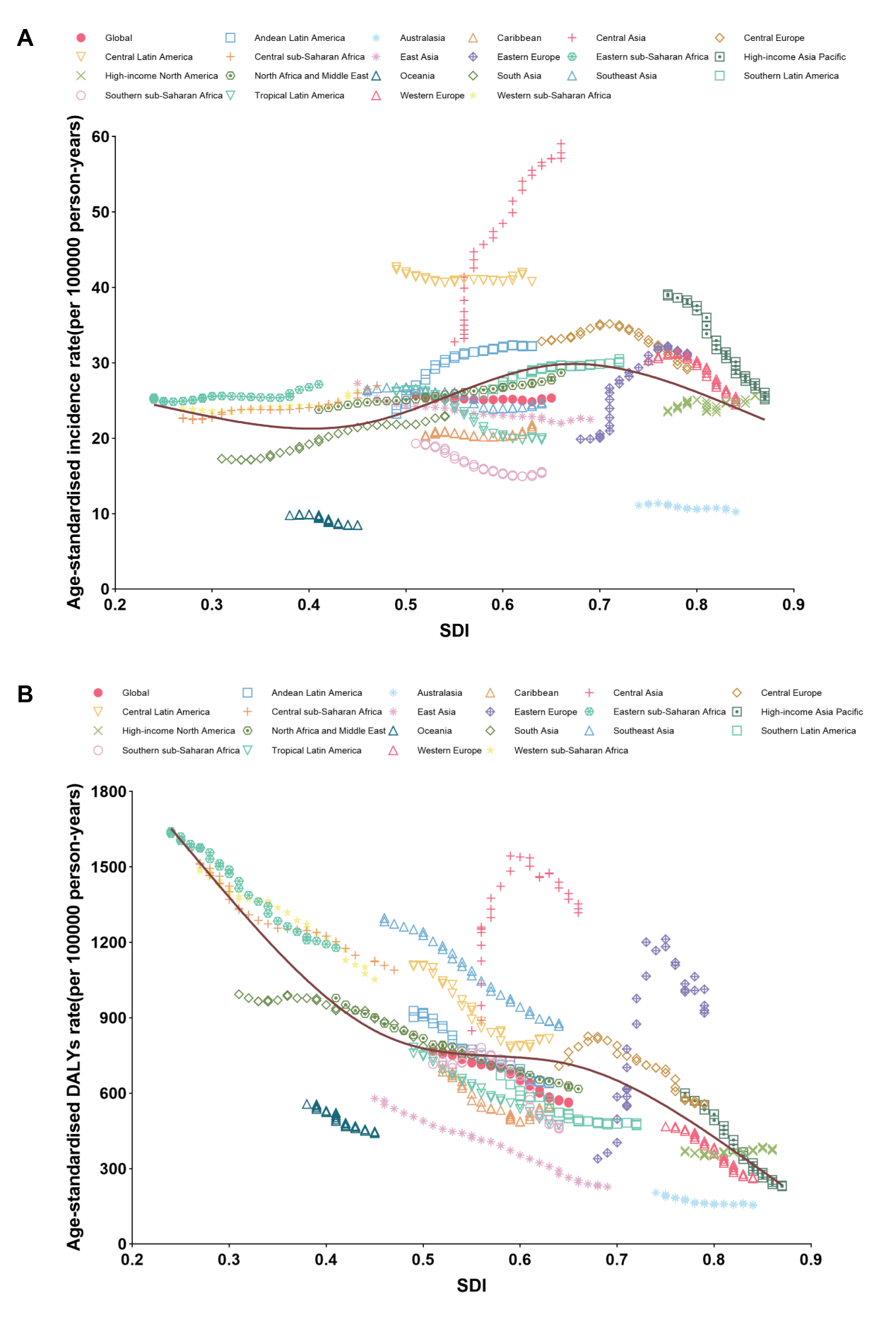
**

Fig.S20：Age-standardized rates of liver cirrhosis and other chronic liver diseases globally and for 21 regions by SDI, 1990-2019 (A) Age-standardized incidence rate per 100 000 population. (B) Age-standardized DALYs rate per 100 000 population.


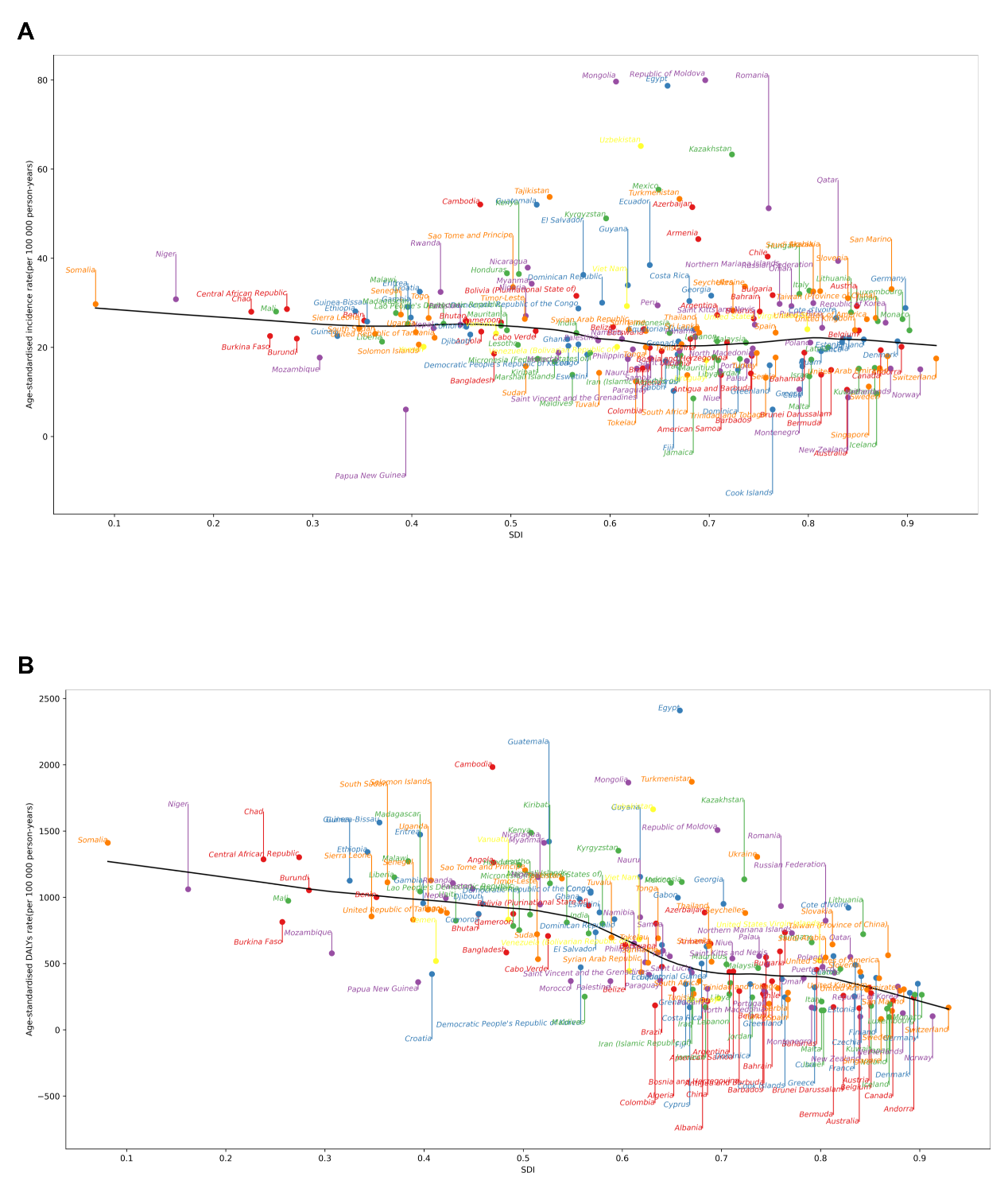


Fig.S21: Age-standardized rates of liver cirrhosis and other chronic liver diseases per 100 000 population by 204 countries and sociodemographic index (SDI), 2019. (A) Age-standardized incidence rate per 100 000 population. (B) Age-standardized DALYs rate per 100 000 population.
